# The historical context and scientific legacy of John O. Holloszy

**DOI:** 10.1152/japplphysiol.00669.2018

**Published:** 2019-02-07

**Authors:** James M. Hagberg, Edward F. Coyle, Kenneth M. Baldwin, Gregory D. Cartee, Luigi Fontana, Michael J. Joyner, John P. Kirwan, Douglas R. Seals, Edward P. Weiss

**Affiliations:** ^1^Department of Kinesiology, University of Maryland School of Public Health, College Park, Maryland; ^2^Department of Kinesiology and Health Education, University of Texas, Austin, Texas; ^3^Department of Physiology and Biophysics, School of Medicine, University of California, Irvine, California; ^4^Muscle Biology Laboratory, School of Kinesiology; Department of Molecular and Integrative Physiology; and Institute of Gerontology, University of Michigan, Ann Arbor, Michigan; ^5^Department of Medicine, Washington University School of Medicine, St. Louis, Missouri; Department of Clinical and Experimental Sciences, Brescia University Medical School, Brescia, Italy; and School of Medicine and Charles Perkins Centre, University of Sydney, Sydney, NSW, Australia; ^6^Department of Anesthesiology and Perioperative Medicine, Mayo Clinic, Rochester, Minnesota; ^7^Pennington Biomedical Research Center, Louisiana State University, Baton Rouge, Louisiana; ^8^Department of Integrative Physiology, University of Colorado Boulder, Boulder, Colorado; ^9^Department of Nutrition and Dietetics, Doisy College of Health Science, St. Louis University, St. Louis, Missouri

**Keywords:** aging, exercise, glucose, skeletal muscle, type 2 diabetes

## Abstract

John O. Holloszy, as perhaps the world’s preeminent exercise biochemist/physiologist, published >400 papers over his 50+ year career, and they have been cited >41,000 times. In 1965 Holloszy showed for the first time that exercise training in rodents resulted in a doubling of skeletal muscle mitochondria, ushering in a very active era of skeletal muscle plasticity research. He subsequently went on to describe the consequences of and the mechanisms underlying these adaptations. Holloszy was first to show that muscle contractions increase muscle glucose transport independent of insulin, and he studied the mechanisms underlying this response throughout his career. He published important papers assessing the impact of training on glucose and insulin metabolism in healthy and diseased humans. Holloszy was at the forefront of rodent studies of caloric restriction and longevity in the 1980s, following these studies with important cross-sectional and longitudinal caloric restriction studies in humans. Holloszy was influential in the discipline of cardiovascular physiology, showing that older healthy and diseased populations could still elicit beneficial cardiovascular adaptations with exercise training. Holloszy and his group made important contributions to exercise physiology on the effects of training on numerous metabolic, hormonal, and cardiovascular adaptations. Holloszy’s outstanding productivity was made possible by his mentoring of ~100 postdoctoral fellows and substantial NIH grant funding over his entire career. Many of these fellows have also played critical roles in the exercise physiology/biochemistry discipline. Thus it is clear that exercise biochemistry and physiology will be influenced by John Holloszy for numerous years to come.

## INTRODUCTION

The last 50 years has seen dramatic advances in the fields of exercise biochemistry and exercise physiology, and a substantial number of these advances have come from the laboratories of Dr. John Holloszy, who spent virtually his entire academic career at Washington University School of Medicine in St. Louis, MO. He began his training as an undergraduate at Oregon State University and then progressed from medical student to resident/intern, research fellow, and through the professorial ranks at Washington University, interrupted only by 2 years at the University of Illinois as a Lt. Commander in the US Public Health Service Heart Disease Control Program.

After initial publications on corticosteroids and renal function in 1961, Holloszy published “The Epidemiology of Coronary Heart Disease: National Differences and the Role of Physical Activity” in 1963 (79). Even though this was only his third publication, it already set the stage for themes that remained prominent over the remainder of his career—exercise, health, and aging. By the middle 1960s there was mounting evidence that exercise or high levels of occupational physical activity were protective against the development of cardiovascular (CV) disease (129). While assigned to the Physical Fitness Laboratory at Illinois to study the endurance training programs pioneered by T. K. Cureton and in collaboration with Jim Skinner, Holloszy published three papers in 1964 on the effects of endurance training on fitness, body composition, blood lipids, and cardiac function in middle-aged men (88, 89, 166), providing some of the first data on these responses to exercise training. After these initial research efforts, and now back at Washington University, in 1965 his seventh paper, with H. T. Narahara, showed that contractions in frog muscles resulted in increased glucose uptake that was independent of insulin (85). His tenth publication, in 1967, remains perhaps his most notable, showing that endurance exercise training resulted in substantial mitochondrial adaptations in rodent skeletal muscle (78).

Together these early papers highlight *1*) the “cutting edge” in the 1960s for many key biological and biomedical questions related to exercise, *2*) the subsequent trajectory for this broad-based topic, and *3*) the future areas of focus for the Holloszy laboratory and his ~100 postdoctoral trainees over the subsequent 50 years. That the topics of his earliest papers are still at the forefront of exercise-related biological and biomedical research is a testament to Holloszy’s early insights. It is also fair to say that he is perhaps the initial proponent of inactivity-induced “mitochondrial dysfunction” as the final common pathway for a host of metabolic and CV disorders (100). His foundational work also demonstrates the power of a single investigator leading a program that combined both human and animal research paradigms. His early use of rodent models in exercise studies is also impressive in this context.

The 1960s was also a time when there was substantial interest in the structural CV adaptations to exercise, extending from functional (vs. pathological) cardiac hypertrophy in response to training and also improved coronary artery function. Again, Holloszy’s early studies address these issues, and the subsequent work in his laboratory from the late 1970s and early 1980s showed just how robust these responses were in younger and older humans and also patients with stable coronary artery disease.

Another major focus of Holloszy’s career was the societal implications of aging and the role of physical activity in blunting age-related physiological declines. Starting in the 1970s the Holloszy laboratory group began pioneering studies on older athletes who served as “experiments in nature” to define the minimum rates of physiological aging (70). This approach was subsequently used to study caloric restriction in humans (49). Additionally, the cross-sectional “experiments in nature” in special populations were then followed by longitudinal intervention studies in humans and animals, including some key early studies on sex differences and the responses of older humans to exercise training (112).

At the dawn of Holloszy’s career in the 1960s, exercise-related research was just starting to move beyond a focus on CV function to a more integrated view of how exercise training or high levels of habitual physical activity influenced CV function and CV disease risk to include a focus on both systemic metabolic adaptations and also adaptations specific to skeletal muscle. A series of superb studies by Holloszy, his collaborators, and trainees defined much of this effort and extended it to aging, CV disease, and also diabetes.

## EFFECT OF EXERCISE TRAINING ON RODENT SKELETAL MUSCLE ADAPTATIONS

Skeletal muscles collectively comprise the organ system of movement. A pivotal property of this system lies in its plasticity, defined as the muscle’s ability to change its mass, metabolic properties, and contractile phenotype in response to alterations in physical activity levels and types. In the early 1950s, investigators used comparative biology approaches to assess muscles of normally active animals versus those that are less active, finding that active animals generally have higher levels of oxidative enzymes in their leg muscles (116, 140). These observations also provided some initial evidence that physical activity differences might contribute to at least a portion of the marked difference in the metabolic properties across different muscle fibers. The first studies to examine the effects of exercise training on skeletal muscle oxidative capacity used a swimming paradigm in rats. Two independent studies in 1956 and 1959 found that 30 min of daily swimming for 5–8 wk did not induce elevations in oxidative enzymes in the leg muscle of rodents (58, 68). In retrospect, this should have not been surprising since later studies showed that rodents could swim for as long as 6 h. Thus these early studies did not generate a sufficient exercise stress, as the activity intensity stimulus was well within the endurance limits of the normally active limb muscles.

**Fig. 1.**
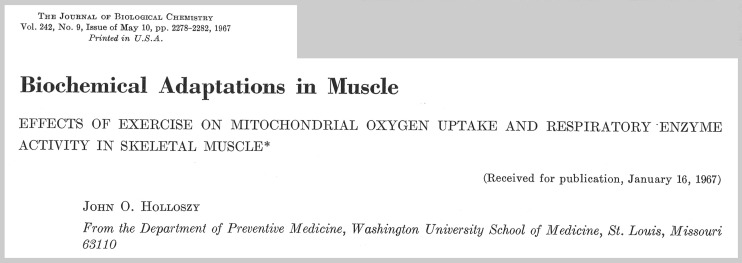
Reprint cover sheet from Holloszy’s original 1967 article in the *Journal of Biological Chemistry* on the adaptations of rodent skeletal muscle to exercise training (*J Biol Chem* 242: 2278–2282, 1967). [Reproduced from Holloszy (78) with permission from the American Society for Biochemistry and Molecular Biology.]

Then in a classic 1967 study Holloszy (Fig. 1) demonstrated that high-intensity running (in contrast to swimming) for progressively longer durations up to 2 h/day, 5 days/wk for 12 wk, nearly doubled the oxidative capacity, respiratory enzyme activity, and mitochondrial biogenesis in rodent limb muscles (Table 1) (78). Additionally, he showed that mitochondria of exercised animals exhibited a high level of respiratory control and a tightly coupled oxidative phosphorylation profile. And perhaps even more importantly, Holloszy’s findings stimulated a markedly enhanced interest in subsequent muscle plasticity studies.

**Table 1.** Gastrocnemius skeletal muscle comparisons between sedentary and exercise-trained rodents

Variable	Sedentary	Exercising
Oxygen uptake, μl·h^−1^·g^−1^	506 ± 53	1,022 ± 118*
P:O ratio	2.7 ± 0.2	2.6 ± 0.1
Cytochrome oxidase activity, μl O_2_·min^−1^·g^−1^	305 ± 15	551 ± 31*
Succinate oxidase activity, μl O_2_·min^−1^·g^−1^	73 ± 5	117 ± 8*
DPNH dehydrogenase activity, μM·min^−1^·g^−1^	5.6 ± 0.6	11.8 ± 1.5*
Mitochondrial protein, mg/g	2.97 + 0.20	4.67 ± 0.30*

Table 1 remade from Tables I, II, and III in Holloszy (78) with permission from the American Society for Biochemistry and Molecular Biology.

* *P* < 0.01, sedentary vs. exercising.

In 1971, Paul Molé in the Holloszy laboratory (128) reported that homogenates from trained rodent skeletal muscle also had a twofold greater capacity than untrained skeletal muscle to metabolize long-chain fatty acid substrates. These findings were one of the first to demonstrate that skeletal muscle had the capacity to use fatty acid substrates for energy during long-duration exercise. Expanding on these results, in 1972 Ken Baldwin (5) from the Holloszy laboratory assessed the effects of endurance training on the biochemical properties of different rodent skeletal muscle fiber types. In addition to histochemical analyses, they assessed the capacity of the muscles to metabolize different substrates (pyruvate and palmitate, a long-chain fatty acid), as well as quantifying marker oxidative enzymes. Although the histochemical results were similar to those from previous studies, the biochemical assessments of muscle homogenates clearly showed that all three muscle types doubled their oxidative capacities to metabolize substrates (Table 2) and increased cytochrome *c* and citrate synthase activity similarly. Hence, this study demonstrated that all types of skeletal muscle have the ability to increase their oxidative capacity with an appropriate exercise training stimulus.

**Table 2.** Oxidation of labeled pyruvate and palmitate by homogenates of different types of muscles from exercised and sedentary animals

	Quadriceps		Soleus
Group	Superficial, white	Deep, red	
*Pyruvate-2-^14^C oxidation, nmol/min per g*			
Sedentary	96 ± 22 (6)	324 ± 56 (6)	158 ± 25 (5)
Runners	207 ± 42 (6)*	832 ± 149 (6)*	376 ± 116 (5)*
*Palmitate-U-^14^C oxidation, nmol/min per g*			
Sedentary	5.5 ± 2.5 (6)	40.5 ± 5.6 (6)	23.2 ± 3.2 (5)
Runners	15.6 ± 2.3 (6)*	88.0 ± 16.0 (6)*	48.0 ± 8.7 (5)*

Values are means ± SE. The number of animals per group is given in parentheses. The concentration of labeled pyruvate was 10 mM, while that of palmitate was 0.75 mM. The pyruvate contained ~70,000 dpm/μM; the palmitate contained ~400,000 dpm/μM. [Reproduced from Baldwin et al. (5) with permission.]

* Runners vs. sedentary, *P* < 0.05.

Shortly after this initial demonstration of increased oxidative enzyme levels in rodent skeletal muscle after exercise training, the Holloszy group began investigating the mechanisms underlying these beneficial adaptive responses. In an initial report postdoctoral fellow Ron Terjung, using a radiolabeled molecule incorporated into the heme proteins of cytochrome *c*, in 1973 reported that the increased levels of cytochrome *c* after training were the result of decreased cytochrome *c* degradation rates (174). Then in 1977 postdoctoral fellow Frank Booth and Holloszy used a training and detraining study design to show that the half-time of the cytochrome *c* response to both training and detraining was 6–8 days in oxidative muscle fiber types (10), which they interpreted as evidence of a training-induced increase in cytochrome *c* synthesis, which has been consistently shown in numerous studies since then.

In a 1973 study the Holloszy group demonstrated that the various skeletal muscle fiber types differentially expressed glycolytic enzymes (6). Furthermore, these enzymes were affected differently by endurance exercise training, with the slow-type fibers increasing their glycolytic capacity while the opposite occurred in the fast-white and fast-red muscle types. These findings collectively demonstrated a pattern of adaptation such that endurance-trained skeletal muscle took on properties more similar to those of cardiac muscle, which represents the epitome of endurance-trained muscle.

In the studies highlighted above and numerous others since then, it was conclusively shown that one of the hallmark adaptations to endurance training is an increase in the density of skeletal muscle mitochondria and, hence, a greater capacity to oxidize substrates. This adaptation is reflected across all skeletal muscle fiber types. Further studies were then conducted to determine the impact that this adaptation played in enhancing exercise capacity. First, in 1973 Baldwin et al. (6) studied rodents that were trained to sustain moderate-intensity running for up to 2 h in duration. Then, subgroups were subsequently run for either 15, 60, or 120 min using three different intensity paradigms typically used in training rodents. One of the regimens involved interval sprint exercise, which also was of higher intensity than the other two regimens. The findings were quite surprising, as only the fast, high-oxidative and slow-oxidative fiber types appeared to contribute to the sustained activity across the three different intensity paradigms, as little evidence indicated that the low-oxidative fast type IIb fibers were recruited. Both oxidative fast and slow fibers markedly depleted their glycogen storage pool, yet only the fast-oxidative fibers utilized significant quantities of stored muscle triglycerides (TG). Interestingly, liver glycogen utilization was extensive across all three exercise paradigms, and at the end of 120 min ~85% of the liver glycogen stores were depleted. In absolute terms, calculations showed that liver glycogen contributed more calories for the exercise than those coming from skeletal muscle glycogen.

In a similar study, Robert Fitts et. al. (46) in 1975 studied rats that were treadmill-trained 5 days/wk for different training session lengths (10, 30, 60, 120 min/session) for 13 wk. They found that the longer the training sessions were, the greater was the increase in oxidative capacity of the muscles. Then a moderate-intensity endurance run to exhaustion and a 30-min test of moderately-high intensity were performed to compare trained versus nontrained groups. Results of the run to exhaustion indicated that there was a progression of running time improvement that correlated with the degree of muscle oxidative capacity, with the animals with the longest training sessions having the greatest running time to exhaustion. In the 30-min endurance run test, the utilization of both muscle and liver glycogen were inversely proportional to the oxidative capacity of skeletal muscle of the group. Thus these studies clearly established the importance of skeletal muscle oxidative capacity in regulating the ability of individuals to exercise until exhaustion by utilizing fuels other than carbohydrate sources.

In subsequent studies by David Wright in the Holloszy group in 2007 (190, 191), several key advances were achieved relative to understanding the mechanisms underlying exercise-induced mitochondrial biogenesis. First, the Holloszy team showed that exercise training-induced mitochondrial biogenesis occurs before there is any upregulation of the PGC-1α gene, suggesting that other mechanisms are operating in the induction process (191). Using an in vitro model of mitochondria biogenesis in C2C12 muscle cells, the Holloszy group showed that p38 mitogen activator protein kinase (p38-MAPK) is first activated and, in turn, it phosphorylates PGC-1α, which is primarily located in the cytosol. This phosphorylation process enables PGC-1α to enter the nucleus to become a transcription coactivator. A second paper by the Holloszy group (190) demonstrated that p38-MAPK phosphorylates and activates activating transcription factor 2 (ATF2), which interacts with MEF2 on the PGC1α promoter to activate its transcription. Thus the role of PGC-1α in mitochondrial biogenesis is biphasic, involving an initial phase of phosphorylation/translocation and then a later phase that elevates PGC-1α levels via increased transcriptional regulation. These studies were pivotal because they demonstrated the molecular bases underlying the plasticity evident in both skeletal muscles in response to chronic exercise.

Over a career that spanned close to 60 years, Holloszy moved the science of exercise and skeletal muscle metabolism into the current cellular and molecular era. His unparalleled intellectual scientific contributions prepared the discipline for the current “-omics” epoch, and now the NIH is embarking on the largest undertaking in the field of exercise and human health through the Molecular Transducers of Physical Activity Consortium (MoTrPAC). This endeavor includes the collection of thousands of human muscle biopsy samples before and after a standardized exercise training intervention and will use current state-of-the-art genomic, proteomic, transcriptomic, and metabolomic approaches to create an exercise-derived molecular roadmap in healthy humans. Such a project is possible because of the prescient thinking and pioneering discoveries of scientific giants such as John Holloszy.

## EXERCISE EFFECTS ON GLUCOSE TRANSPORT IN SKELETAL MUSCLE OF ANIMAL MODELS

In addition to his groundbreaking studies on the adaptations of skeletal muscle to exercise training, Holloszy also published numerous paradigm-shifting studies on the effects of contractile activity or exercise on glucose uptake by skeletal muscle in animal models.

### Effects of Exercise/Contractile Activity on Insulin-Independent Glucose Uptake by Skeletal Muscle

Research in the 1950s provided evidence for effects of contractile activity on glucoregulation, but these experiments relied on the measurement of circulating glucose levels, which could not directly assess the role of skeletal muscle (57, 97, 98). Helmreich and Cori (72a) and Sacks and Smith (160) reported in rats and cats, respectively, that electrically induced contractions led to accumulation in skeletal muscle of pentoses originating from the circulation, but this approach could not distinguish between extramuscular (e.g., blood flow or other systemic factors) and intramuscular mechanisms. These experiments laid the foundation for one of Holloszy’s earliest and most influential publications (85). He and his postdoctoral mentor H. T. Narahara quantified the accumulation of radiolabeled 3-*O*-methylglucose, a nonmetabolizable glucose analog that had recently become available, in isolated frog sartorius muscles that were electrically stimulated to induce contractions. Their results clearly established for the first time that contractile activity acted directly on skeletal muscle to increase hexose transport independent of circulating insulin, altered blood flow, or posttransport glucose metabolism. Kinetic experiments revealed that contractile activity increased *V*_max_ without altering the apparent *K*_m_, leading them to conclude that the greater glucose transport was the result of an “increase in the number of operative sites for sugar transport,” a very prescient statement in 1965.

**Fig. 2.**
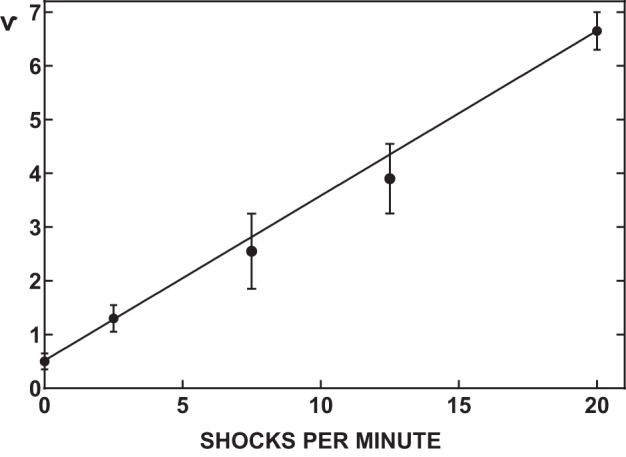
Relationship of permeability to the frequency of stimulation in isolated frog muscles. Muscles of winter frogs were stimulated at 19° at various frequencies, and the plateau of permeability attained after 2 h is presented. Each point indicates the mean of 4 muscles, and vertical bars represent twice the SE. [Reproduced from Holloszy and Narahara (85) with permission from the American Society for Biochemistry and Molecular Biology.]

The research by Holloszy and Narahara was groundbreaking because they aimed to elucidate the relationship between increased glucose transport and the complex series of molecular events required for muscle contraction, including membrane depolarization, release of sarcoplasmic reticulum Ca^2+^ into the cytosol, actin and myosin interactions, ATP hydrolysis, and other metabolic processes (83, 84). After their initial study described above, they assessed the effect of incubating frog muscles with caffeine at doses sufficient to increase cytosolic Ca^2+^ and cause contractions without depolarizing the sarcolemma (83). They showed that membrane depolarization was not essential for contraction-stimulated glucose transport. They then manipulated mechanical work during contractions and found no relationship between the glucose transport rate and the amount of work performed or the metabolic challenge based on muscle lactate or creatine phosphate levels (85). In contrast, a linear relationship was observed between the frequency of electrical stimulation and the rate of glucose transport (Fig. 2). Because contraction frequency was known to directly relate to cytosolic Ca^2+^ concentration, these results supported their hypothesis that Ca^2+^ was part of the mechanism for contraction-stimulated glucose transport. Consistent with this idea, including graded amounts of extracellular Ca^2+^ in the incubation media along with potassium to induce contraction resulted in progressively greater ^45^Ca^2+^ accumulation and 3-*O*-methylglucose transport (83).

In the 1970s it was proposed that a “permissive” amount of insulin was required for contraction-stimulated glucose transport (8). Working with Holloszy, Harriett Wallberg-Henriksson et al. (179, 180) used streptozotocin-induced diabetic rats and extensively washed perfused or isolated muscle to remove all residual insulin before demonstrating that glucose uptake could still be increased by contraction. Ploug et al. (142) from Copenhagen also found that insulin was not required for the contraction effect on muscle glucose uptake of diabetic rats perfused with insulin antiserum.

Studying frog muscle, Holloszy and Narahara found that a combination of maximally effective insulin and contractile activity did not produce greater increases in glucose uptake than either stimulus alone (85). In contrast, Nesher et al. (132) in isolated rat muscles and Zorzano et al. (196) in perfused rat hindlimb muscles reported additive effects of insulin and contractile activity on glucose uptake. Steve Constable in the Holloszy laboratory found similar results in the isolated rat epitrochlearis muscle (21a). The consistency of these results in rat muscle is clear, but the reasons for the difference between rat and frog muscles are uncertain.

In the 1980s, Cushman (29) and Suzuki and Kono (172) independently provided evidence for the “translocation hypothesis” (i.e., redistribution of glucose transporter proteins from intracellular membranes to the plasma membrane) to explain the rapid, insulin-mediated increase in glucose transport in adipocytes. There was great interest in testing this concept in skeletal muscle with insulin and exercise/contractile activity. After Wardzala used isolated rat diaphragms to demonstrate insulin-stimulated glucose transporter translocation in skeletal muscle (181), Holloszy and Amira Klip from Toronto collaborated to elucidate the influence of insulin and exercise/contractile activity on glucose transporter translocation in skeletal muscle. Their approach used differential centrifugation and sucrose gradients to purify muscle membrane fractions enriched with the sarcolemma or internal membranes. A study by Andre Douen from the Klip group along with the Holloszy group revealed that both in vivo insulin treatment and acute exercise resulted in greater GLUT4, but not GLUT1, glucose transporter protein in cell surface membranes in rat skeletal muscle (38). Jiaping Gao with Holloszy (54) reported greater cell surface GLUT4 content with either insulin or contractile activity alone. Furthermore, muscles stimulated with both insulin and contractile activity had greater GLUT4 content than each independent stimulus. These results were consistent with the finding that combined insulin and contractile activity have an additive effect on glucose uptake. Research by others using alternative approaches, including GLUT4 photolabeling by Lund et al. (118) or microscopy with fluorescently tagged GLUT4 by Lauritzen et al. (115), supported the idea that in rat muscle, insulin and contractile activity produce additive or near additive effects on cell surface GLUT4.

The Holloszy group performed additional experiments using rat skeletal muscle to further evaluate the putative role of cytosolic Ca^2+^ in contraction-stimulated glucose transport. Jang Youn et al. (195) reported that doses of the compound W-7 which stimulate sarcoplasmic reticulum (SR) release of Ca^2+^ without inducing measurable tension development by isolated rat muscles resulted in greater glucose transport. W7 did not alter ATP or creatine phosphate concentration. Furthermore, dantrolene, an inhibitor of SR Ca^2+^ release, eliminated the effect of W7 on glucose transport.

In 1996, Will Winder, then at BYU after being a fellow and faculty member with Holloszy, and Graham Hardie demonstrated that exercise increased AMP-activated protein kinase (AMPK) activity in rat skeletal muscle (187). The following year, Winder’s laboratory discovered that 5-aminoimidazole-4-carboxamide ribonucleoside (AICAR) could stimulate AMPK activity and glucose uptake in perfused rat skeletal muscle (124). David Wright in the Holloszy laboratory evaluated the independent and combined effects of caffeine and AICAR on glucose transport in isolated rat epitrochlearis muscles (192). Glucose transport was increased by a caffeine dose insufficient to induce tension development. AICAR also increased glucose transport, and the simultaneous incubation of muscles with caffeine and AICAR induced greater glucose transport than either stimulus alone. They hypothesized that contraction-stimulated glucose transport may be due to the combined actions of Ca^2+^ and AMPK. Research from a number of researchers has suggested that, in addition to possible roles for these Ca^2+^- and AMPK-related processes, the complex mechanism for exercise/contraction-stimulated glucose transport may involve a variety of other cellular events, e.g., tension development, nitric oxide production, and activation of the sucrose-nonfermenting AMPK-related kinase (119, 130, 149).

### The Effects of Acute Exercise on Subsequent Insulin-Stimulated Glucose Uptake

In 1982, Erik Richter working in Neil Ruderman’s laboratory published a study using perfused rat hindlimbs demonstrating for the first time that acute exercise could lead to a subsequent increase in insulin-stimulated glucose uptake by skeletal muscle (148). To follow up on these findings John Ivy in the Holloszy laboratory assessed glucose uptake in perfused rat muscle after acute and chronic exercise. He concluded that the most recent training bout before measuring glucose uptake (i.e., acute exercise) appeared largely responsible for the benefits of chronic exercise on insulin-stimulated glucose uptake (99).

Presumably an event that occurs during exercise initiates the subsequent increase in insulin-stimulated glucose uptake that can last for many hours following the conclusion of the exercise. AMPK activation, a hallmark of exercise in the recruited muscle, is an attractive candidate for this initial event. Supporting this idea, Jon Fisher in the Holloszy group found that incubating isolated rat muscles with the AMPK activator AICAR led to a subsequent increase in insulin-stimulated glucose uptake (45). Jørgen Wojtaszewski’s group recently reported that prior incubation with AICAR can also lead to greater insulin-stimulated glucose uptake by isolated mouse muscles (111). This effect was absent in AMPK-deficient mice, consistent with AMPK’s putative role as a trigger for subsequently increased insulin sensitivity.

**Fig. 3.**
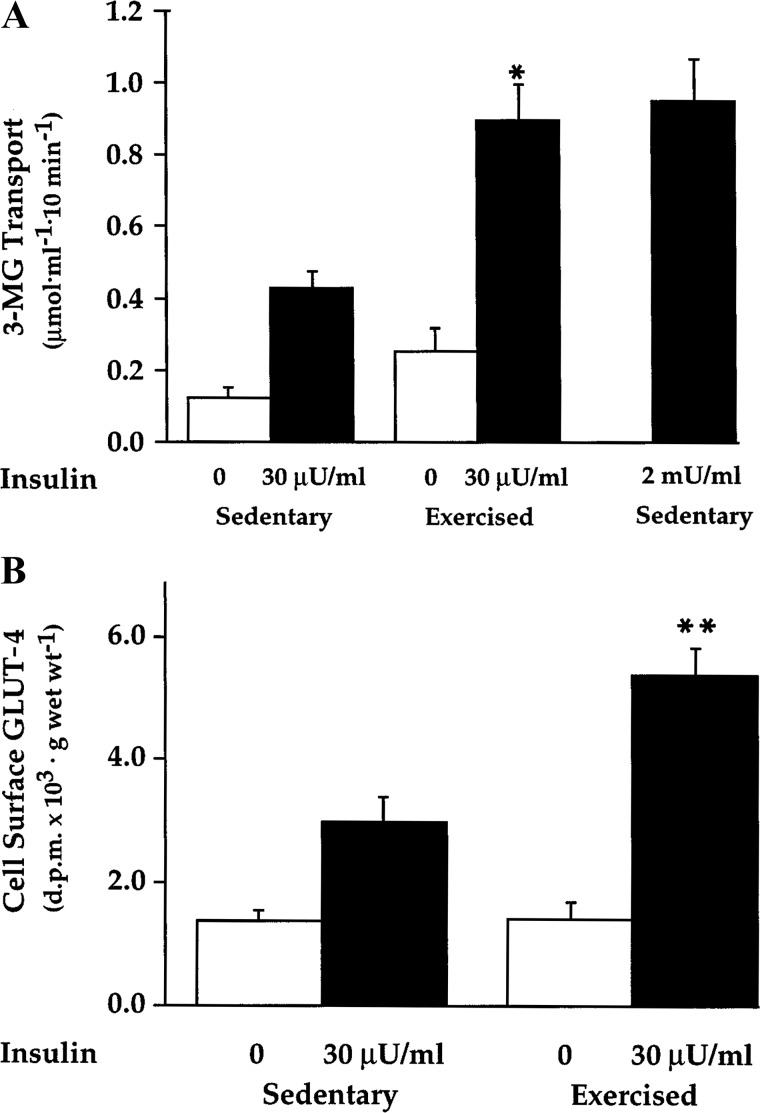
Effect of exercise on insulin sensitivity of glucose transport and cell surface GLUT-4 levels in rat epitrochlearis muscle. Rats were exercised by swimming for 2 h. Epitrochlearis muscles were incubated in vivo for 3 h and then were further incubated with the indicated concentration of insulin before measurement of 3-MG transport or cell surface GLUT-4 content. Data are means ± SE for *n* = 5 muscles/group (3-MG transport) or 10 (GLUT-4 concentrations). *P < 0.01, **P < 0.001 for exercised vs. sedentary. [Reproduced from Hansen et al. (66) with permission.]

The Holloszy group also provided insights into the roles of proximal insulin signaling and GLUT4 translocation in the postexercise increase in insulin sensitivity. Polly Hansen reported that the greater insulin-stimulated glucose uptake 3.5 h after acute exercise was accompanied by a proportional increase in cell surface-localized GLUT4 with unaltered total GLUT4 abundance (Fig. 3) (66). Improved insulin sensitivity occurred without amplifying tyrosine phosphorylation of the insulin receptor or insulin receptor substrate-1 in insulin-stimulated muscles. These results support those of Wojtaszewski et al. (189) and others who found greater insulin sensitivity after acute exercise without amplification of proximal insulin signaling steps in rodent or human muscle (13).

Also in the Holloszy laboratory, Paige Geiger evaluated the influence of incubating isolated rat epitrochlearis muscles with a supraphysiological insulin concentration, removing the insulin for several hours, and then exposing the muscles to a submaximally effective insulin concentration (56). After this treatment, the muscles were more insulin sensitive. They hypothesized that after GLUT4 are initially recruited to the cell surface membranes by various stimuli (e.g., exercise, AICAR or insulin), the GLUT4 is internalized to an unidentified intracellular “high susceptibility compartment” where the GLUT4 is more easily recruited to the cell surface in response to a subsequent stimulus. Testing this provocative hypothesis awaits the refinement and application of methods that enable tracking of the localization of specific GLUT4 molecules over several hours.

In addition to identifying the mechanisms responsible for initiating the increase in insulin sensitivity, it is also important to understand the processes that reverse the effect. Research from the Holloszy laboratory showed that the increased insulin-stimulated glucose uptake in isolated muscles after acute exercise could persist for up to 18–48 h in rats that were fed a carbohydrate-free diet after exercise (14). When rats were provided with their typical high-carbohydrate chow diet following the same exercise, increased insulin sensitivity was reversed more rapidly and concomitant with glycogen supercompensation.

### Effects of Acute or Chronic Exercise on GLUT4 Glucose Transporter Expression in Skeletal Muscle

Erik Henriksen in the Holloszy group found that skeletal muscle GLUT4 abundance correlated closely with glucose transport capacity (73). Increased GLUT4 glucose transporter abundance is now recognized as a hallmark of chronic exercise training (149). This observation was first reported in 1990 by several independent groups. Ken Rodnick working with Holloszy and David James (153) found that GLUT4 protein abundance in plantaris muscles was greater after 6 wk of chronic exercise compared with controls. In a collaboration between the laboratories of Lynis Dohm and Mike Sherman, Friedman et al. reported that muscle GLUT4 protein abundance was increased above controls after 18 or 32 wk of endurance training in obese Zucker rats (51). Ploug et al. reported that 10 wk of exercise training in rats elevated both GLUT4 protein levels and maximal insulin-stimulated glucose uptake of fast-twitch red gastrocnemius (143). Similarly, in 1992, Rodnick et al. found that the training-induced increase in epitrochlearis GLUT4 was accompanied by a proportionally greater insulin-stimulated glucose uptake (152). Jian-Ming Ren from the Holloszy group found that long-term exercise training was not required to increase muscle GLUT4 protein. He found that 16 h after a prolonged exercise session (2 bouts of 3 h separated by 45 min rest), GLUT4 mRNA, GLUT4 protein, and insulin-stimulated glucose transport markedly exceeded values from muscles from sedentary rats (147).

In 1999, Will Winder’s group reported that injecting rats for 5 consecutive days with the AMPK activator AICAR resulted in increased GLUT4 protein abundance in skeletal muscles (91). Shortly thereafter, Ed Ojuka in the Holloszy group probed AMPK and other mechanisms regulating GLUT4 protein expression in skeletal muscle. His first study using isolated rat muscles incubated with AICAR for 18 h found that GLUT4 protein abundance was increased to a similar extent as found in rat muscles 18 h after prolonged exercise (2 bouts of 3 h separated by 45 min rest) (138). In another study, he found that either AICAR (3 h per day for 5 days) or caffeine (3 h per day for 5 days) increased GLUT4 protein levels and insulin-stimulated glucose uptake in L6 cells (137). Caffeine’s effect on GLUT4 abundance was eliminated in caffeine-treated cells that were simultaneously exposed to dantrolene, which reduced caffeine’s effect on cytosolic Ca^2+^, or KN93, a calmodulin-dependent protein kinase inhibitor. The GLUT4 promoter contains a MEF2 binding site that engages MEF2A-MEF2D heterodimers, and incubation of isolated rat muscles with either caffeine or ionomycin (a Ca^2+^ ionophore) for 18 h resulted in greater abundance of MEF2A and MEF2D concomitant with increased GLUT4 protein levels.

Keith Baar in the Holloszy group found that transgenic mice overexpressing the transcription factor nuclear respiratory factor 1 (NRF-1) were characterized by increased muscle expression of MEF2A and GLUT4, as well as a greater capacity for insulin-stimulated glucose uptake (4). He subsequently reported that a single prolonged exercise session (2 bouts of 3 h separated by 45 min rest) resulted in greater NRF1 and NRF2 protein abundance at either 12 or 18 h postexercise (3). Mark Hargreaves’ group in Australia, Ed Ojuka’s group in South Africa, and others have also made important findings regarding the mechanisms for exercise-induced GLUT4 expression (149).

If one considers only Holloszy’s work using animal models to understand glucose transport in skeletal muscle, his accomplishments would represent an impressive research career. He convincingly demonstrated that contractile activity alone is sufficient, independent of insulin or other systemic factors, to increase glucose transport in skeletal muscle. His collaborative work with Amira Klip’s group revealed that acute exercise’s effects on insulin-independent glucose transport are attributable to redistribution of GLUT4 from the cell’s interior to its surface membranes. His laboratory demonstrated that insulin and contraction have additive effects on glucose transport and GLUT4 localization to the cell surface. His group also discovered that the enhanced insulin-stimulated glucose transport several hours after acute exercise is secondary to greater GLUT4 recruitment to the cell surface. His research was instrumental in establishing that increased GLUT4 protein abundance in skeletal muscle is a hallmark of chronic exercise, and a series of studies from his laboratory made significant contributions to the current understanding of the molecular processes responsible for this adaptation. Finally, his discoveries using animal models paved the way for his laboratory’s important research on the effects of exercise on glucose metabolism in humans.

## HUMAN GLUCOSE METABOLISM STUDIES

In Western society, the typical human gains weight and engages in less physical activity as they age. This type of sedentary lifestyle causes obesity and a host of metabolic changes that lead to type 2 diabetes and CV disease. Holloszy was among the first to recognize that physical exercise could arrest and even reverse these conditions, and since the 1960s his research program has provided the foundation that has helped shape our current understanding of how exercise modulates the body’s molecular, cellular, and physiological systems, and provides a vital tool to counter chronic diseases.

Exercise is now widely recommended as one of the first management strategies for patients with abnormal glucose metabolism and, together with diet and behavior modification, is a central component of all type 2 diabetes and obesity prevention and treatment programs. It is known that adults who maintain a physically active lifestyle have significantly reduced risk of developing impaired glucose tolerance, insulin resistance, and type 2 diabetes (109). The Holloszy laboratory was among the first to describe the benefits of exercise on glucose regulation in humans. One of his earliest reports was a 1977 study with Finn Gyntelberg examining the effect of exercise training on the glucoregulatory hormones, insulin and glucagon (59). The training program was standardized based on relative exercise intensity and was performed 4 days/wk for 10 wk. The exercise training was effective as evidenced by the 18% increase in maximal oxygen uptake (V̇o_2max_). They also found that glucagon increased while insulin decreased during exercise, and notably that exercise training attenuated both of these responses. These results were complementary to the growing literature on hormonal regulation during exercise that was being generated from Europe, primarily in the Christensen laboratory in Copenhagen (52, 53, 148). Together, these studies contributed significantly to our understanding of human physiology, but it was the adaptive responses to exercise training rather than the acute response to exercise that caught Holloszy’s attention and led to a series of novel and pioneering studies that unraveled many of the effects of exercise training on glucose metabolism in humans.

**Fig. 4.**
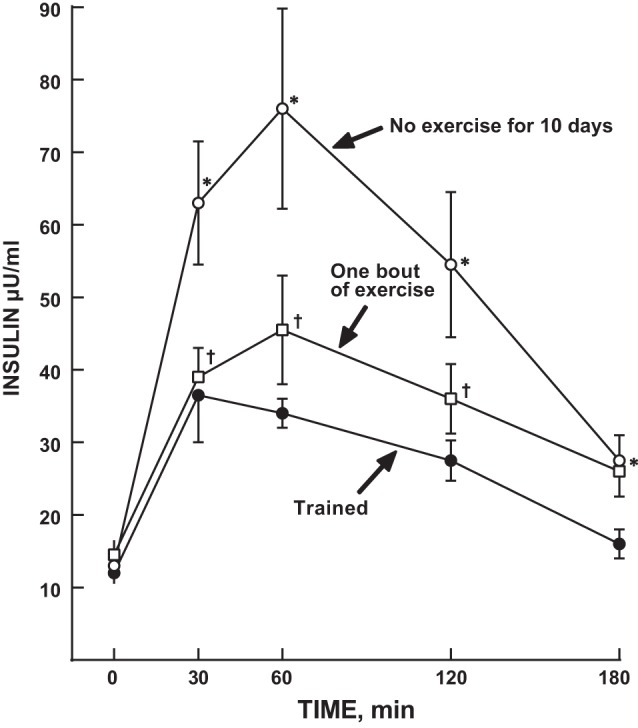
Effect of stopping exercise for 10 days followed by one bout of usual exercise on plasma insulin responses to an oral glucose tolerance test (OGTT) in young trained men and women. Points are means SE for 8 subjects. *Trained vs. No exercise for 10 days, *P* < 0.05. †One bout of exercise vs. No exercise for 10 days *P* < 0.05. [Reproduced from Heath et al. (69) with permission.]

His next human studies in hormonal regulation of glucose metabolism moved away from examining static insulin and glucagon responses to exercise and focused instead on glucose tolerance and insulin sensitivity. At the time, some data indicated that physically trained individuals had normal glucose responses when challenged with an oral glucose tolerance test (OGTT). However, this occurred in the presence of a noticeably lower insulin response, which gave rise to the idea that exercise training resulted in increased insulin sensitivity. It should be noted that “insulin sensitivity” during this time period was defined on the basis of plasma glucose and insulin responses during an OGTT. At first the increased insulin sensitivity was attributed, by association, to a higher V̇o_2max_ and metabolic capacity and a leaner phenotype. However, building on his earlier observations of the acute effect of exercise on insulin in rodent models, Holloszy posited that the residual effect of the prior single bout of exercise was generating this effect. To test this, postdoctoral fellow Greg Heath measured OGTT glucose and insulin responses in a group of highly trained individuals who agreed to stop exercising for 10 days (69). Aerobic capacity and body fat did not change during this period and, thus, any response could not be attributed to changes in metabolic capacity or body composition. However, following 10 days of no exercise their initially markedly reduced insulin response to glucose was eliminated and was virtually the same as that of sedentary individuals of the same age (Fig. 4). And, despite higher insulin levels, their OGTT glucose concentrations were also slightly increased. Participants then performed a single bout of exercise, and the OGTT glucose and insulin responses were assessed again. Now their OGTT insulin (Fig. 4) and glucose responses were similar to those evident when they were training regularly, suggesting that much of the benefit of exercise training on glucose regulation and insulin sensitivity is due to metabolic and cellular adaptations generated during single bouts of exercise which persist for only a relatively short period.

Building on these results, the Holloszy laboratory then further explored the effect of exercise training on glucose metabolism in older men and women and in patients with type 2 diabetes, where glucose tolerance and insulin resistance are highly prevalent and clinically problematic. Their initial studies compared older individuals who had trained most of their life (masters athletes—“experiments in nature”) with age-matched individuals who had not exercised (87, 162, 163). In all cases, glucose tolerance and insulin sensitivity were enhanced in those who exercised, and indeed the older masters athletes appeared to have successfully overcome the age-related glucose intolerance and deterioration in insulin sensitivity. However, whether these age-related benefits were due to training per se, i.e., long-term physiological and metabolic adaptations, or to the residual effects of the last bout of exercise was unclear. Using the 10-day no-exercise model previously employed by Heath et al. (69), Marc Rogers showed that for most masters athletes the effect was due to adaptation and protection against the age-related development of glucose intolerance (155). This conclusion was based on the generally similar glucose and insulin responses to glucose ingestion in the masters athletes after 10 days without exercise compared with healthy young controls who had not participated in a regular exercise program. However, the response was not uniform and some of the participants experienced a decline in glucose tolerance even to the level of being prediabetic. These results also highlighted the variability in human responses to exercise and the need to maintain a lifelong exercise training program for those most susceptible to glucose intolerance and potentially type 2 diabetes.

**Fig. 5.**
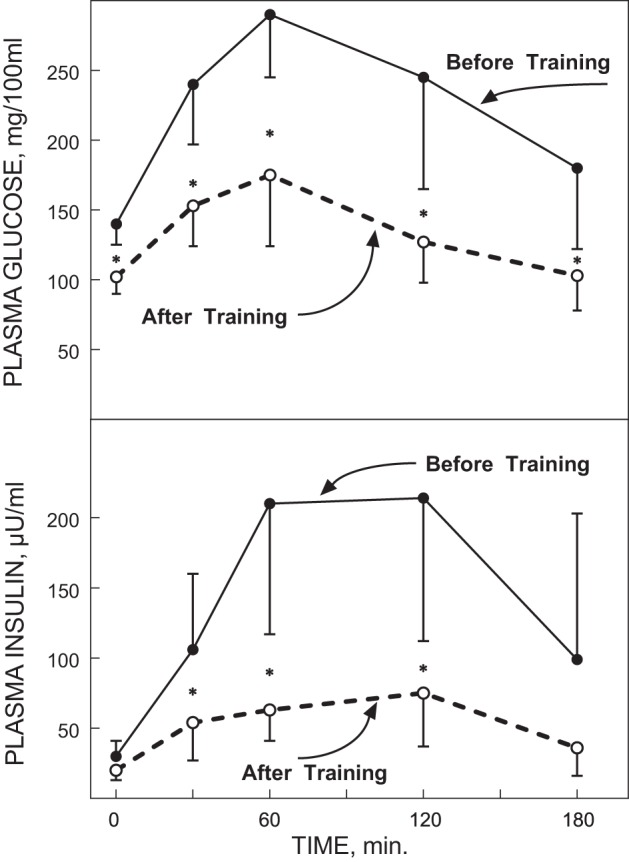
Plasma glucose (*top* panel) and insulin responses (*bottom* panel) to an oral glucose tolerance test (OGTT) in 5 type 2 diabetes patients before and after a 12-mo intense exercise training intervention. *P < 0.01. [Reproduced from Holloszy et al. (87) with permission from John Wiley. Copyright 2009 John Wiley & Sons.]

**Fig. 6.**
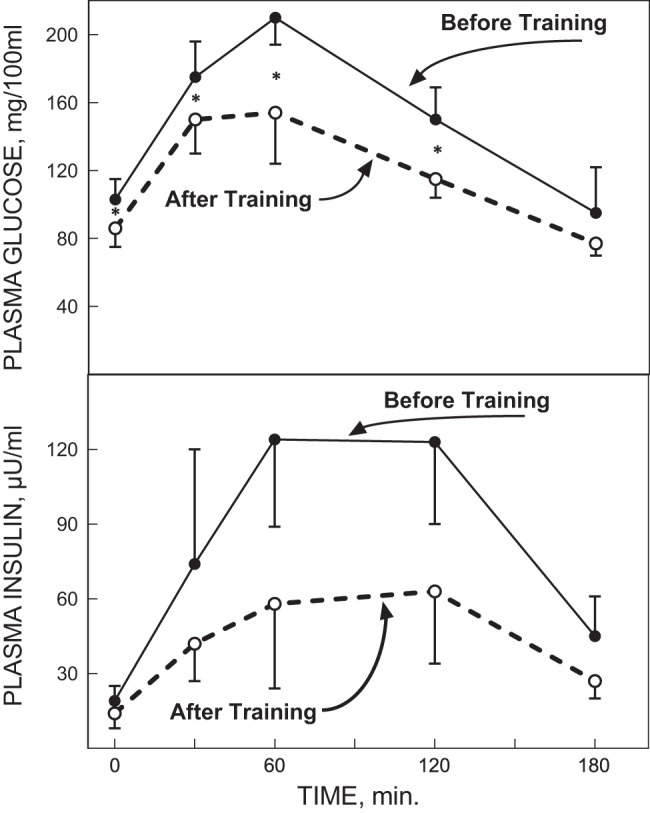
Plasma glucose (*top* panel) and insulin responses (*bottom* panel) to an oral glucose tolerance test (OGTT) in 8 individuals with impaired glucose tolerance before and after a 12-mo intense exercise training intervention. *P < 0.01. [Reproduced from Holloszy et al. (87) with permission from John Wiley. Copyright 2009 John Wiley & Sons.]

The next obvious question then clearly was if exercise training could reverse the insulin-resistant state in individuals with type 2 diabetes. In a classic 1986 paper Holloszy and his group quantified the effects of a 1-yr, 5 day/wk, 1 h/session exercise program that progressed to high-intensity training for the last 6 mo in a small group of CV disease patients (87). Five of the men initially presented with type 2 diabetes, but after training, three of them no longer showed a diabetic OGTT response (Fig. 5). Similarly, eight of the men initially had impaired glucose tolerance and all eight normalized their OGTT glucose responses with training (Fig. 6). These marked improvements in OGTT responses were associated with ~50–80% reductions in OGTT insulin responses. However, it is important to note that these men lost an average of 4–5 kg of body weight with the exercise training program, as a direct result of the increased energy expenditure associated with their exercise as they did not alter their caloric intake over this time. Thus these results clearly showed that a prolonged relatively intense exercise training program combined with moderate weight loss could markedly improve, even “cure,” the diabetic and prediabetic state of a number of these CAD patients. However, mechanistically the question arises as to whether the improved glucose metabolism was the result of the exercise training or the weight loss and also whether it actually took the entire year of exercise training to elicit these marked improvements. Marc Rogers in the Holloszy laboratory then addressed this further in an elegantly designed study that allowed them to test the effect of exercise training without the prolonged exercise training program or the confounding effects of changes in body composition (157). They studied a group with mild type 2 diabetes before and after a 7-day aerobic exercise training program. With the 7 days of exercise both glucose tolerance and insulin sensitivity improved significantly, whereas V̇o_2max_ and body composition were unchanged. But then, was the improvement simply due to the last bout of exercise? This was addressed by also measuring OGTT glucose and insulin responses after the initial single bout of exercise. However, the single bout of exercise did not alter glucose tolerance or insulin sensitivity, perhaps because these individuals simply could not do very much exercise in a single session as a result of their very low exercise capacities. In these landmark human physiological studies, Holloszy and his team were among the first to demonstrate that exercise training produces adaptive physiological and metabolic responses that have therapeutic potential for the prevention and treatment of glucose intolerance.

Holloszy was always at the forefront of advances in technology and procedures. In 1979, Ralph DeFronzo, Jordan Tobin, and Rubin Andres published a seminal paper in the *American Journal of Physiology* that dramatically improved the methods to assess peripheral insulin sensitivity and secretion in humans (32). The “glucose-clamp technique” provided quantitative data on pancreatic insulin secretion using an infusion of glucose (hyperglycemic clamp), and a measure of skeletal muscle insulin sensitivity, by simultaneously infusing insulin and glucose (euglycemic insulin clamp). These methodologies opened up new opportunities to evaluate the role of exercise in preventing “insulin resistance,” a term that is now ubiquitously used to describe the aberrant glucose metabolism associated with type 2 diabetes and aging. Insulin sensitivity, which can be viewed as the inverse of insulin resistance, is evaluated in terms of the concentration of insulin required to induce 50% of its maximal effect on glucose transport (101). An increase in insulin sensitivity causes the insulin dose-response curve to shift to the left, and consequently the insulin concentration required to cause 50% of its maximal effect is lower. Insulin responsiveness, in contrast, determines the magnitude of the increase in glucose transport induced by a maximally effective insulin stimulus (101). An increase in insulin responsiveness is the result of a larger increase in glucose transport in response to a maximal insulin stimulus and a proportional upward shift in the insulin dose-response curve.

### Role of Exercise in Clamp-Derived Insulin Sensitivity and Responsiveness

Gail Dalsky, Doug King, and Myrlene Staten were the first to establish these complex and challenging methods in the Holloszy laboratory (with some of the authors of this paper being the initial participants!). Between 1987 and the early 1990s they examined the effects of exercise on insulin sensitivity and responsiveness, and on insulin secretion, in young and older trained and untrained men and women. Using a two-stage hyperinsulinemic euglycemic clamp, they found that endurance-trained men and women had increased insulin sensitivity, but similar responsiveness to insulin, compared with untrained individuals (104). Further, when the trained subjects refrained from exercising for 10 days, insulin sensitivity was significantly reduced compared with the trained state, but insulin responsiveness remained unaffected (102). These observations were also noted by Dela and colleagues (34, 35), and have now been confirmed by numerous laboratories.

**Fig. 7.**
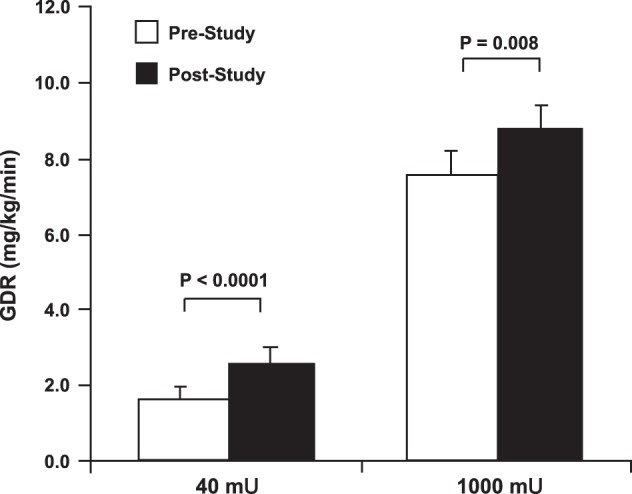
Effects of a 7-day exercise training program on insulin sensitivity and insulin responsiveness. GDR, glucose disposal rate. [Reproduced with Kirwan et al. (110) with permission.]

Holloszy subsequently proposed that exercise training might increase insulin sensitivity and responsiveness assessed with these newer and more valid methods in type 2 diabetes. Marc Rogers from the Holloszy laboratory had previously reported improved glucose tolerance after just 7 days of exercise training (157), and so a similar exercise training study was performed in individuals with type 2 diabetes, but using a two-stage hyperinsulinemic (40, 1,000 mU·m^−2^·min^−1^) euglycemic clamp and an isotopic glucose tracer to measure insulin sensitivity and responsiveness, and hepatic glucose production, respectively (110). The average rate of glucose disposal during the low-dose insulin infusion was 24% of that attained during the maximal insulin stimulus (Fig. 7). After training, the rate of glucose disposal during the low-dose stage was 30% of that attained during the maximal insulin stimulus. Thus, while the actual rate of glucose disposal during the 40 mU·m^−2^·min^−1^ insulin dose was 45% higher after training, the rate of glucose disposal expressed relative to the maximal rate was only actually 25% higher as the training also elicited a 17% increase in insulin responsiveness. These results established that in addition to increased insulin sensitivity, an increase in insulin responsiveness also plays an essential role in the improvement in insulin action induced by short-term exercise training in patients with type 2 diabetes.

### Effect of Exercise on Clamp-Derived Insulin Secretion

**Fig. 8.**
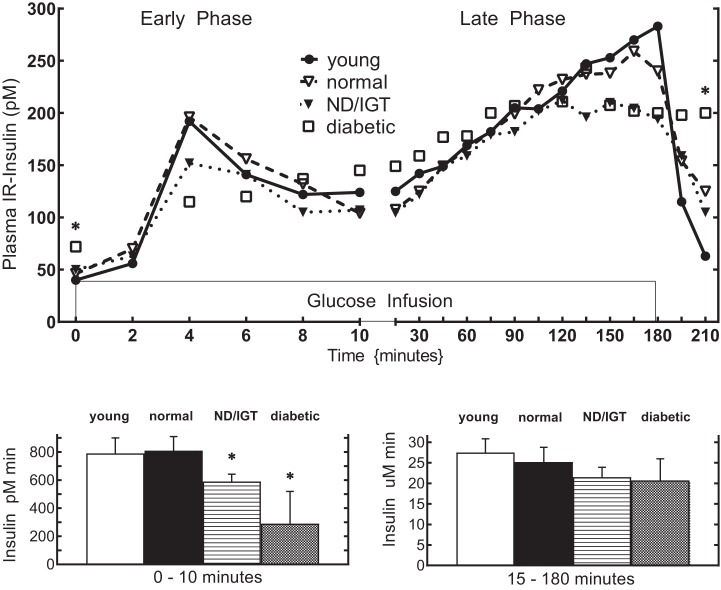
Plasma insulin responses during a hyperglycemic clamp and a 30-min recovery period, divided to show the early and late phases. The bottom panels represent the incremental insulin area above baseline. Mean ± SE are presented in the bar graphs. *Different from young (*P* < 0.05). ND/IGT, nondiagnostic/impaired glucose tolerance. [Reproduced from Bourey et al. (11) with permission from Oxford University Press. Copyright 1993 Oxford University Press.]

Insulin resistance in adipose tissue, muscle, or the liver places greater demand on insulin secretion from pancreatic beta cells. For many, this hypersecretory state is unsustainable, and the subsequent loss of beta cell function marks the onset of type 2 diabetes (15). Fasting plasma glucose, insulin, and glucagon levels are generally poor indicators of beta cell function. The Holloszy laboratory was among a vanguard group who used the OGTT and hyperglycemic clamp techniques to more accurately measure the dynamic regulation of glucose homeostasis by the pancreas (Fig. 8) (11). These and related studies reported that under conditions of controlled hyperglycemia, when the glucose stimulus to the beta cell is identical, exercise-trained individuals have markedly lower first- and second-phase insulin responses compared with untrained age- and weight-matched individuals (104). This effect was also shown to be independent of other insulin secretagogues such as arginine or a high-fat meal (105). To assess whether the effect of training on insulin secretion was an enduring feature of the trained state, Doug King and colleagues detrained a group of younger athletes for 14 days (103). Again, using the hyperglycemic clamp, they found that both early- and late-phase insulin secretion was significantly increased in these individuals after the period of inactivity. Similar observations were noted by Mikines and colleagues around the same time (126). These data highlighted how exercise effects on physiological function in young trained individuals are relatively short lived and served to reinforce the notion that the benefits of exercise on insulin action require adoption of a lifelong exercise training program performed most if not all days of the week.

John Kirwan of the Holloszy laboratory then tested the effects of exercise training on glucose-stimulated insulin secretion in individuals with known reductions in secretory capacity (108). In this study 65- to 70-yr-old men and women completed a 12-wk aerobic exercise training program. Insulin secretion was assessed before and within 24 h of the last exercise bout using a hyperglycemic clamp. As hypothesized, exercise training reduced both first- and second-phase insulin responses, by magnitudes of 13% and 23%, respectively. Based on prior observations that the effects of exercise training are relatively short-lived, one could argue that these effects are mostly due to the last bout of exercise. Although this is possible from a mechanistic viewpoint, it is not necessarily a clinically useful distinction. If untrained individuals, particularly those older or obese, try to perform a single bout of exercise that is close, or equivalent, to what they can perform after exercise training, they are likely to induce muscle damage. Indeed, Kirwan and Holloszy demonstrated that an acute bout of exercise that would produce a comparable amount of work as could be performed after exercise training does indeed cause muscle damage and induces skeletal muscle insulin resistance and an increase in insulin secretion in young and older adults (106, 107, 113). This led to the conclusion that for the most part, the reduced insulin secretory response that is observed in the exercise-trained state is transitory, but to achieve and sustain the effect, one must exercise vigorously and to an extent that is only possible through a consistent and persistent exercise training program.

As it became clear that exercise alone had unique and specific effects on insulin secretion, attention turned to the question of what additional effects, if any, could be achieved with exercise alone as compared with diet alone. To address this, Paul Arciero and colleagues in the Holloszy laboratory examined the individual effects of 10 days of exercise or low-calorie (50% reduction in total calorie intake) diet on insulin secretion using a combination of hyperglycemic clamp, arginine infusion, and high-fat drink to probe insulin secretory capacity in obese prediabetic men and women (1). The study revealed that while both interventions significantly lowered the insulin response to glucose, the 56% increase in glucose uptake after exercise compared with the 19% increase after the low-calorie diet suggested that exercise may be more effective than diet in correcting metabolism in the presence of obesity and insulin resistance.

### Mechanisms Regulating Insulin Action and Glucose Metabolism in Humans

A major insulin-independent regulator of glucose uptake is the fuel-sensing enzyme AMPK. Will Winder, one of the early postdoctoral fellows in Holloszy’s laboratory, was among the first to elucidate the role of exercise-induced AMPK activation in skeletal muscle (67, 187). It is noteworthy that AMPK remains transiently activated after exercise and regulates several downstream targets involved in mitochondrial biogenesis and function and oxidative capacity (159). Molecular control of muscle mitochondrial content and function is controlled by two master transcriptional regulators, peroxisome proliferator-activated receptor-γ coactivator (PGC)-1α and -1β (PPAR α and PPARβ, respectively). One of the most recent novel findings the Holloszy laboratory found is that PPARβ increases PGC-1α by protecting it from degradation. PPARβ also induced an increase in mitochondrial respiratory chain proteins and MEF2A, through nuclear respiratory factor 1 (NRF-1). Knockdown of PPARβ resulted in large decreases in PGC-1α and mitochondrial proteins and a reduction in exercise-induced mitochondrial biogenesis. This study opened a new area of research on how mitochondrial content and function may be regulated, and identified PPARβ as an essential nuclear factor in the exercise-mediated response to exercise.

Over his career John Holloszy dramatically advanced our understanding of the mechanisms by which exercise improves glucose metabolism and insulin resistance by studying the biochemical complexities of skeletal muscle metabolism to reveal a rich tapestry of interconnected signaling pathways that control and regulate cellular glucose homeostasis. Holloszy paved the way for true translational science that began with observations in human physiology and in animal models, then used in vitro cellular approaches that identified mechanistic control points that were then translated forward using intervention studies to elicit improvements in physiological and metabolic function in insulin-resistant humans. The insights gained from his career of research in the area of human glucose and insulin metabolism alone are clearly very impressive and have changed the science in this discipline in terms of basic biochemical and physiological mechanisms, as well as the clinical treatment of patients with type 2 diabetes.

## CALORIE RESTRICTION AND AGING

Another scientific discipline where Holloszy made substantial contributions is with respect to calorie restriction (CR) and aging. CR refers to the minimization of energy intake while meeting the needs for other essential nutrients. Over four decades Holloszy has published numerous seminal papers that advanced our understanding of the effects of CR on aging. During the early half of this period his work focused on animal studies to evaluate theories on aging and to differentiate the lifespan-extending effects of CR, per se, from those which might be attributable to lower body weight and less energy availability. Subsequently, his focus moved to human studies to determine if CR affects aging in humans, as it does in animal models.

### Calorie Restriction in Rodents

By the early 1980s, numerous animal studies had demonstrated that CR increases both average lifespan (i.e., mean age at death, resulting from all causes of death, for all animals in the group) and maximal lifespan (age at death of the oldest individuals in a group, reflective of primary aging—the basic biological clock that keeps ticking, even in the absence of overt disease). However, because CR results in less energy availability and lower body weight when compared with free-feeding control groups, it was not clear if this lifespan-extending effect of CR was due to CR itself or to low energy availability and low body weight. To address this, Holloszy, Susan Garthwaite, and colleagues compared rats undergoing CR to those who maintained the same body weight through voluntary wheel running, and to a sedentary, free-feeding control group (55) and showed that CR and wheel running both increased average lifespan versus controls, but only CR increased maximal lifespan. This was taken as evidence that CR slows primary aging processes, independent of the effects of low body weight and low energy availability.

**Fig. 9.**
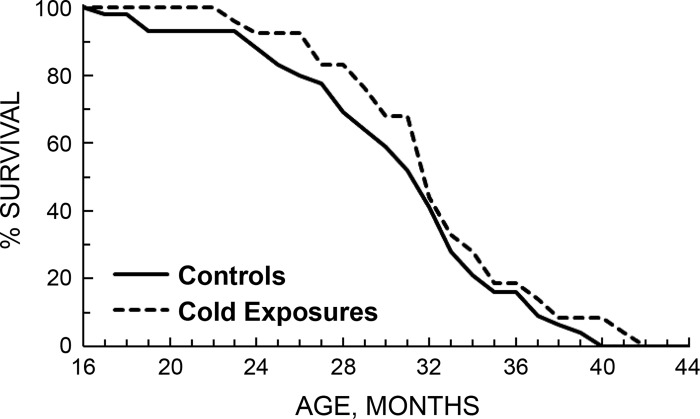
Survival curves of control rats compared with those who underwent cold exposure to assess the “rate of living” theory of aging. [Reproduced from Holloszy and Smith (90) with permission.]

Holloszy recognized that an alternative explanation for his finding that CR, but not exercise, increases maximal lifespan is that exercise might have negative effects on maximal lifespan, thereby counteracting the beneficial effects of low energy availability and low body weight. This was an especially valid concern because the “rate of living” theory (33), which postulated that increases in energy expenditure accelerate aging, had not yet been disproven. To address this issue he exposed rodents to cool water several days per week to increase lifelong energy expenditure and compared their lifespan to that of control rats (90). Average and maximal lifespan were not different from that in the control rats, suggesting that elevations in energy expenditure do not accelerate aging (Fig. 9). Therefore, exercise-induced increases in energy expenditure would not likely offset the beneficial effects of low energy availability and low body weight, if such effects existed.

**Fig. 10.**
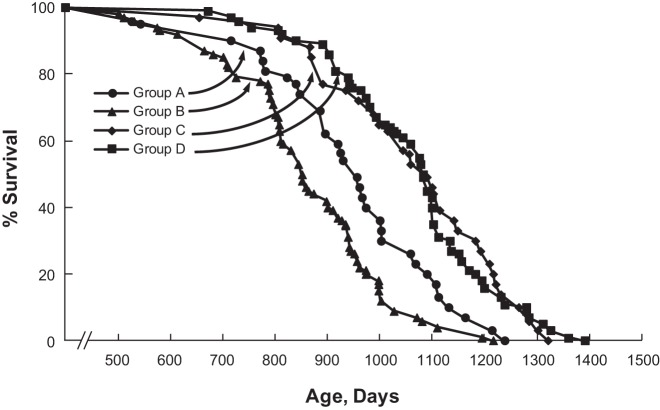
Survival curves for four groups: *group A*: runners; *group B*: sedentary controls; *group C*: food-restricted runners; and *group D*: food-restricted sedentary controls. Survival curve for sedentary control rats in group B is significantly different from that of runners in group A (*P* < 0.02), food-restricted runners in group C (P < 0.0001), and food-restricted sedentary rats in group D (*P* < 0.0001). Survival curve for runners in group A is significantly different from that of food-restricted runners in group C (*P* < 0.01) and food-restricted sedentary rats in group D (*P* < 0.01). [Reproduced from Holloszy (80) with permission.]

Holloszy used another experimental approach to evaluate the possibility that exercise might counteract the effects of low energy availability and low body weight on primary aging by comparing rodents undergoing a combination of CR and exercise to weight-matched rats undergoing only CR (80, 86). Results showed that average and maximal lifespan were similar in the CR plus exercise group as in the CR alone group, and both were greater than in a sedentary, free-feeding control group (Fig. 10). This provided further evidence that exercise does not have negative effects on aging and that it does not interfere with the beneficial effect of CR on lifespan.

Holloszy’s landmark studies outlined above provided several advances in our knowledge about the effects of CR on aging. First, CR increases maximal lifespan while similarly low energy availability and low body weight induced by exercise do not; this suggests that CR-related factors other than low energy availability and low body weight are responsible for the maximal lifespan-extending effects of CR. Second, demonstrating that increased energy expenditure induced by cold exposure does not negatively affect lifespan suggests that increases in energy expenditure from exercise would not be likely to counteract any beneficial effects of low energy availability and low body weight on lifespan, if such effects existed. Last, further evidence that exercise does not counteract the beneficial effects of CR comes from the studies which showed that CR plus exercise increases maximal lifespan to a similar extent as CR alone. Together, these findings indicated that the effects of CR on primary aging and maximal lifespan are not mediated by low energy availability or low body weight. Thus these studies were instrumental in setting the course for subsequent research efforts to understand the mechanisms by which CR alters primary aging.

### Calorie Restriction in Humans: Research Strategies

From a scientific perspective, there is great interest in elucidating the mechanisms that regulate aging in all animal species, and CR clearly provides a useful tool for advancing this scientific agenda. However, part of the rationale for studying CR in animals is to gain insights about how CR might be used to slow aging in humans. Definitive randomized intervention studies to determine if CR affects maximal lifespan in humans are not possible for several scientific and logistical reasons. Despite these barriers, Holloszy and his team made great advances by using three strategies to further our understanding of the effects of CR in humans. First, they identified biological signatures of CR that have commonly been observed in animal studies and determined if they are present in CR humans. Second, based on the premise that CR but not exercise slows primary aging and increases maximal lifespan, Holloszy’s studies often included a weight-matched exercising comparison group. Last, they sought to measure biological functions that change predictably with increasing age, independent of the presence or absence of frank disease (for example, cardiac diastolic function gradually decreases over the adult lifespan, even in the absence of cardiac disease).

### Calorie Restriction in Humans: Observational Studies of Long-Term, Self-Imposed Calorie Restriction

Starting in the early 2000s Luigi Fontana and Holloszy collaborated to conduct several observational studies of individuals undergoing self-imposed, long-term, strict CR with optimal nutrition. CR practitioners, “experiments in nature,” from the US and other countries traveled to St. Louis, MO, to participate in these studies. Most were middle-aged (50 ± 10 yr) men who had been practicing CR for 3–15 yr and were weight stable (49). To ensure adequate intakes of micronutrients, the CR practitioners focused on consuming micronutrient-rich, low-energy-density foods, such as nonstarchy vegetables, with many also self-monitoring micronutrient intakes. As in the rodent CR studies, most of these studies included an exercising comparison group that was matched to the CR group for age, sex, and body mass, and a nonobese control group consuming a Western diet and performing little or no exercise.

A key strategy was to evaluate CR biomarkers that had been identified in animal studies to determine if they are also altered in humans undergoing CR. One of the adaptations thought to contribute to the effect of CR on aging is an energy-conserving reduction in metabolic rate. In animals, this appears to be mediated by reductions in triiodothyronine (T3) (74, 158). Indeed, subclinically low serum T3 levels were observed in CR practitioners (48). Importantly, alterations in other thyroid hormone levels were not observed, suggesting that this was not a state of thyroid dysfunction, but rather that it was an adaptive metabolic response to conserve energy. Animal studies have also shown decreases in body temperature in response to CR (39, 150). In the human studies performed by Fontana, Holloszy, and Soare, daytime, nighttime, and 24-h average core temperatures were lower in CR practitioners than in controls (167). The same phenomenon was not observed in exercisers who were matched to the CR group for sex, age, body weight, and fat mass. These findings are in line with Holloszy’s theory that CR, but not exercise, slows primary aging and extends maximal lifespan.

**Fig. 11.**
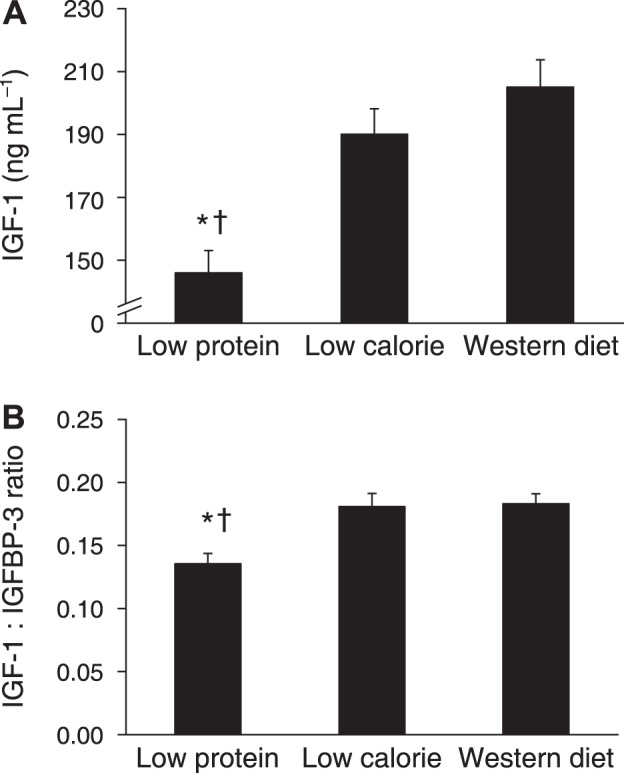
Long-term effects of caloric restriction and protein restriction on serum IGF-1 concentration (*A*) and the ratio of IGF-1 to IGFBP-3 concentrations (*B*). Data from the cross-sectional comparison of individuals who were habitually consuming a low-protein diet, a low-calorie diet, or a typical Western diet. Data are means SE. *P ≤ 0.01 vs. the low-calorie group. †*P* ≤ 0.01 vs. the Western diet group. [Reproduced from Fontana et al. (50) under Creative Commons Attribution License 4.0.]

Other biomarkers of CR that were identified in animal studies include reductions in growth factors, which in addition to having effects on primary aging, may also decrease cancer development (40, 168). Fontana, Holloszy, and Cangemi found lower levels of several bioavailable growth factors in CR practitioners, including insulin-like growth factor 1 (IGF-1), sex hormones, and insulin (12, 47). No such alterations were observed in lean exercisers. This work also provided insights about the effect of dietary protein on CR-induced changes in growth factors and cancer risk. Many of the CR practitioners had relatively high intakes of protein (more than double the RDA) and as a result, IGF-1 levels were not different than in the control group (50) (Fig. 11). However, in a small intervention study, the CR practitioners reduced their protein intake to the RDA and this decreased their IGF-1 levels (50), suggesting that high protein intakes may interfere with some of the physiological adaptations to CR. Further studies of these CR practitioners have provided mechanistic insights about the effect of CR on primary aging and cancer risk in humans by showing that cellular quality control processes such as apoptosis and autophagy are enhanced by CR (194).

**Fig. 12.**
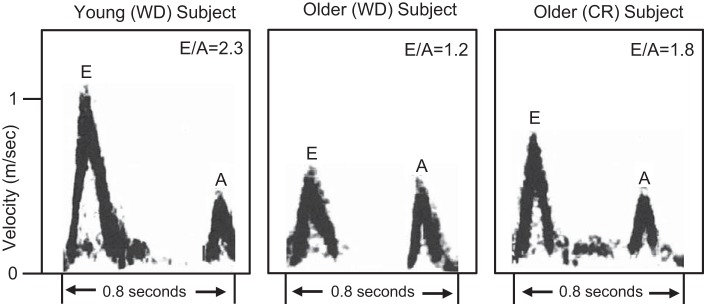
Doppler mitral valve inflow patterns for a typical young healthy individual on a Western diet (WD), an older individual on a Western diet (WD), and an older caloric-restricted (CR) individual. [Reproduced from Meyer et al. (125) with permission from Elsevier Inc. Copyright The American College of Cardiology Foundation.]

Fontana and Holloszy devised a complementary strategy to study CR and aging in humans by identifying physiological measures that change with advancing age, even in the absence of disease. The two primary physiological functions they studied were cardiac diastolic function and autonomic control of heart rate. Tim Meyer, Fontana, Holloszy, and colleagues found that, compared with controls, CR practitioners had better diastolic function as evidenced by motion analysis of the mitral valve annulus (125) (Fig. 12). Analysis of ventricular filling kinetics showed that this effect was mediated by less ventricular stiffness and less viscoelastic impediment to ventricular filling. These effects of CR were accompanied by lower serum levels of transforming growth factor β, suggesting less myocardial fibrosis. Autonomic control of heart rate, as determined from heart rate variability (HRV) measures, is also known to deteriorate with increasing age (175). As compared with age- and sex-matched control subjects, CR practitioners had greater HRV, with values that were comparable to those that would be predicted for healthy individuals who were 20 years younger (170). This finding is suggestive of less age-related deterioration in autonomic sympathetic and parasympathetic system function.

In aggregate, by studying individuals who had been practicing strict CR for several years, “experiments in nature,” Fontana, Holloszy, and colleagues published numerous highly influential studies on CR and primary aging in humans. These studies showed that many adaptations to CR that occur in animals also occur in humans. Furthermore, by studying physiological functions that normally deteriorate with advancing age in humans, CR was found to result in a more youthful phenotype. These findings support the notion that CR may slow primary aging in humans as it does in animals.

### Calorie Restriction in Humans: Intervention Studies

Although the aforementioned observational studies of CR practitioners have advantages (e.g., CR exposure is very long term, adherence to CR is high) over intervention studies, observational studies have well-known limitations. Therefore, Holloszy led a large research team (including Susan Racette, Edward Weiss, Dennis Villareal, and Luigi Fontana) to perform human CR intervention studies as part of a multicenter project called CALERIE (Comprehensive Assessment of the Long-term Effects of Restricted Intake of Energy). CALERIE consisted of two phases and ran from 2002 to 2012. During phase 1, which consisted of distinct studies at each of the study sites, Holloszy and his team implemented a randomized intervention trial to compare 1 yr of 20% CR to an exercise group undergoing the same weight loss and same energy deficit without CR; a control group was also included. Adaptations to CR that were also observed in the exercise group were likely attributable to weight loss whereas adaptations that occurred in the CR group only would have been attributable to CR, per se. To be consistent with the methods used in animal studies, CALERIE excluded obese individuals and focused on normal to slightly overweight individuals. Body mass and body fat decreased similarly in the CR and exercise group (8–12% weight loss) and did not change in the control group, indicating the feasibility of performing a year-long CR and exercise intervention in healthy, nonobese adults (144).

Similar to the observational studies of CR practitioners, phase 1 CALERIE included cardiac diastolic function and T3 hormone levels as outcomes that could be used to gain insights about the effects of CR on primary aging. Riordan, Holloszy, and colleagues found that cardiac isovolumic relaxation time decreased and early diastolic filling increased only in the CR group, suggesting improved diastolic function (151). Furthermore, analysis of ventricular filling kinetics revealed reductions in myocardial stiffness also only in the CR group. These findings indicate that CR resulted in a “younger” cardiac phenotype and that this benefit appears to result from CR itself, because the same amount of weight loss induced by exercise did not provide such benefits. Alterations in thyroid hormone concentrations also mirrored the findings from CR in animals as Edward Weiss, Holloszy, and colleagues found that serum T3 levels decreased in the CR group, but not in the exercise or control groups (185). Although CR reduced T3 levels, they remained in the low-normal range and were not accompanied by changes in other thyroid hormones, suggesting again that the changes were not pathologic. Together, these findings from CALERIE phase 1 suggest that CR may slow aging in humans as it does in animals.

Holloszy was also one of the lead investigators for the multi-center CALERIE phase 2 project, which consisted of a common protocol followed by all three study sites. In this study, 218 nonobese, healthy men and women were randomized to 2 yr of 25% calorie restriction (CR) or control (146). The main outcomes were biomarkers of CR that had been observed in animal CR studies. Although intervention adherence was initially good and body weight decreased for 12 mo, the degree of CR diminished to 8% during *year 2* and body weight tended to increase. Resting metabolic rate decreased after 1 yr of CR, but it reverted back to baseline by the end of *year 2*. Core body temperature did not change; however, T3 levels were lower throughout the 2-yr intervention. Taken together, the lower resting metabolic rate during the period of greatest CR compliance and the reduction in T3 hormone levels are consistent with the effects of CR on animals and support the notion that CR may slow primary aging and have beneficial effects on lifespan in humans. However, the difficulty in sustaining strict CR for 2 yr raises serious concerns about the ability of most people to adhere to long-term CR.

### Dehydroepiandrosterone and Aging

Beginning in the late 1990s Holloszy and colleagues began studying the role of dehydroepiandrosterone (DHEA) in aging. DHEA is the most abundant adrenal androgen in humans (2). After peaking at age ~20 yr, plasma DHEA levels decline steadily with advancing age such that adults aged ~70 yr have 80% lower serum DHEA concentrations than young adults (139). Holloszy and others surmised that the remarkable decline in DHEA with increasing age may contribute to the development of age-related disease processes. This logic was partly based on evidence indicating that DHEA is an activator of the nuclear transcription factor PPARα (141), which has known effects on fat metabolism (114) and inflammation (117). Accordingly, the age-related decline in DHEA levels could contribute to increased adiposity and inflammation and their sequelae, such as insulin resistance and CVD. Because DHEA is also a precursor to sex hormones, he also recognized that reductions in DHEA during adulthood could have effects that are mediated by reductions in testosterone and its downstream hormone, IGF-1.

To assess the role of DHEA in the age-related deterioration of physiological function, Holloszy conducted DHEA supplementation studies in humans. His focus was on DHEA doses that restored or preserved “youthful” levels of circulating DHEA during aging. He was not interested in supraphysiological levels that would not be relevant to understanding the role of DHEA in aging. His earliest DHEA studies were on rodents. Along with Dong Ho Han and Poly Hansen, Holloszy showed that DHEA supplementation protected rats that were fed a high-fat diet from the accumulation of visceral fat and the development of insulin resistance, despite no alterations in food intake (65). Furthermore, in the absence of a high-fat diet, DHEA supplementation protected rats against age-related accumulation of adipose tissue and preserved insulin sensitivity and signaling in skeletal muscle (64).

Holloszy subsequently performed numerous randomized controlled trials (RCT) on humans, along with Dennis Villareal and Edward Weiss. In an open-label study, they found that 6 mo of replacement DHEA doses (50 mg/day) in older adults (~70 yr) partially reversed several age-related changes in fat mass and fat-free mass (178). These findings were later confirmed in a double-blinded, placebo-controlled trial (177). In the open-label study, they also found clinically relevant ~2% increases in bone mineral density (BMD) (178); they later confirmed this finding in a larger 2-yr double-blinded RCT, but found the effects to be more specific to women than men (183). Based on the increases in the anabolic hormone IGF-1 in these studies, Holloszy proposed that DHEA replacement therapy might augment adaptations to strength training in older adults, which could have important implications for preventing and treating sarcopenia. Indeed, in a placebo-controlled trial, DHEA replacement augmented the increases in muscle strength and size that occurred in response to 4 mo of resistance exercise training (176).

**Fig. 13.**
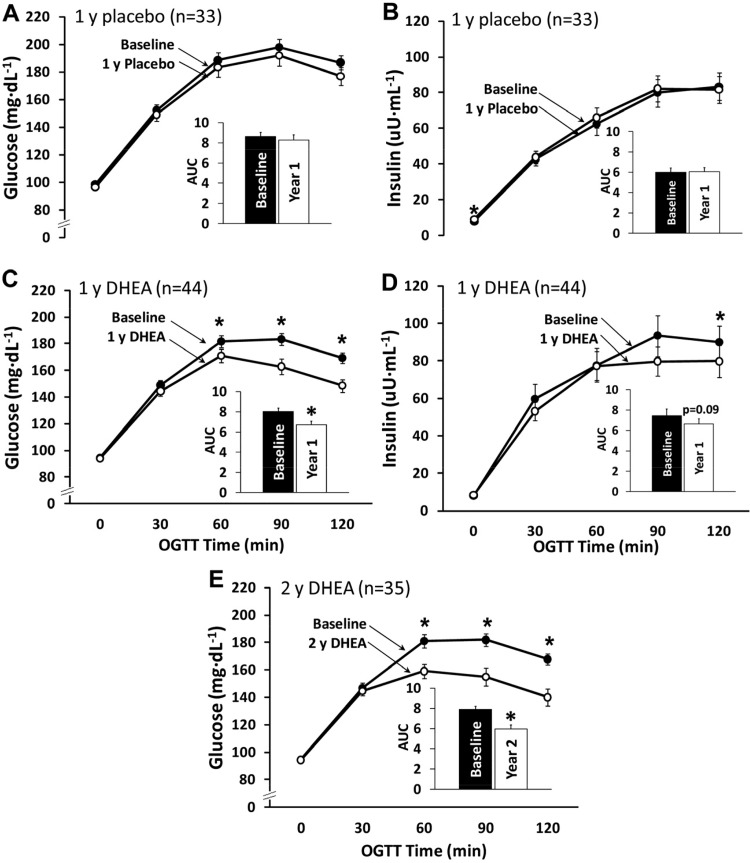
Oral glucose tolerance test (OGTT) glucose and insulin responses for individuals with initial abnormal glucose tolerance in the placebo and DHEA supplementation groups. The reduction in glucose area under the curve (AUC) in the DHEA group (*C*) was significantly greater than that for the placebo group (*A*; *P* = 0.03). Changes in the insulin AUC did not differ between groups (*B* and *D*; *P* = 0.52). Improvements in glucose AUC were maintained in a subset of DHEA group participants who completed a 2nd yr of DHEA supplementation (*E*; *P* < 0.05 for baseline vs. DHEA). *P < 0.05 for baseline versus DHEA. [Reproduced from Weiss et al. (184) under Creative Commons Attribution License 3.0. Copyright Weiss et al. (184).]

In accordance with Holloszy’s earlier studies in rodents, and with the reductions in adiposity and the potential anti-inflammatory effects of DHEA, Holloszy, Villareal, and Weiss also demonstrated that DHEA replacement improves insulin action in older adults. In the earlier open-label study, insulin levels during an OGTT decreased, while glucose levels remained unchanged, in response to DHEA supplementation, suggesting enhanced insulin action. This finding led to a placebo-controlled RCT by Holloszy; at the same time, a similar RCT was initiated by Nair et al. at Mayo Clinic in Rochester. The Holloszy and Nair studies reported contradictory results, with Nair’s group showing no beneficial effect of DHEA on insulin action (131). However, on closer examination, the discrepant findings from the two studies were explained by differences in baseline levels of insulin action, such that DHEA improves insulin action, along with associated reductions in IL6 and TNFα, primarily in subjects who have insulin resistance at baseline (Fig. 13) (184). Additional measures from Holloszy’s double-blinded RCT showed that DHEA also had beneficial effects on the vasculature. Based on reductions in carotid artery augmentation index and carotid-femoral pulse-wave velocity, DHEA replacement resulted in a predicted 20-yr reversal of age-related increases in arterial stiffness. These changes were associated with reductions in inflammatory markers and with increases in free testosterone. Furthermore, based on associations of arterial stiffness with cardiovascular and all-cause mortality, the observed changes would be predicted to reduce mortality risk by ~17%.

During nearly four decades of work, Holloszy produced many important advances in our understanding of the role of CR on aging and longevity. In his early studies he used comparisons of weight-matched rodents undergoing CR versus exercise to show that CR slowed primary aging and increased maximal lifespan through effects that were independent of low energy availability and low body weight. He also generated convincing evidence that the lack of an effect of exercise to increase maximal lifespan in rodents was not a consequence of negative effects of exercise on aging and longevity. Beginning in the early 2000s, Holloszy and his colleagues made significant advances in determining whether CR slows primary aging in humans. Observational studies of individuals undergoing long-term self-imposed CR indicated that CR results in many of the same biological changes in humans as it does in animal studies and also creates a more youthful phenotype. He and his team also showed that these adaptations do not occur in lean endurance athletes, suggesting that CR per se provide these benefits, not leanness. Finally, Holloszy and colleagues performed human intervention trials of CR and generated additional evidence that biological responses to CR in humans appear to mirror the signatures of CR that are evident in animal studies.

In addition, Holloszy and his team made numerous important contributions to our state of knowledge about the physiological effects of DHEA. His contributions demonstrated that DHEA supplementation in older adults has important effects on health, with implications relevant to obesity, osteoporosis, sarcopenia, and cardiometabolic disease. However, these findings also provide insights about the role of DHEA in aging.

## EXERCISE AND CV FUNCTION

Although Holloszy’s research in exercise metabolism is legendary, he and his laboratory also made some critically important contributions to CV exercise physiology, particularly in the settings of aging and patients with coronary artery disease (CAD). This work was part of the Holloszy laboratory human research program, the success of which involved the additional leadership, oversight, and scientific efforts of cardiologist Ali Ehsani and physiologist Jim Hagberg.

To investigate important gaps in this area, they used a highly effective combination of cross-sectional and interventional study designs. Although middle-aged and older masters athletes had been studied somewhat previously (“experiments in nature”), Holloszy used the cross-sectional masters athlete model extensively as a time- and resource-efficient approach to screen for possible long-term effects of vigorous exercise on CV function with aging. The basic model involved comparing groups of young sedentary or exercise-trained adults with corresponding groups of middle-aged and older subjects. The older groups were rigorously screened for CAD and other clinical disorders, which allowed the isolation of the effects of physical activity and aging as much as is possible in free-living human populations.

### Maximal Aerobic Exercise Capacity and Aging

Maximal aerobic exercise capacity (cardiorespiratory fitness) is a critical determinant of overall physical work capacity and one of the most important independent risk factors for future morbidity, functional limitations/disability, and mortality with aging. It was known that maximal aerobic exercise capacity, as assessed by oxygen consumption during maximal exercise (V̇o_2max_), decreased with aging in humans. Holloszy was interested in the role of physical activity and other factors in age-related declines in maximal aerobic exercise capacity, and the mechanisms by which these influences exerted their effects.

**Fig. 14.**
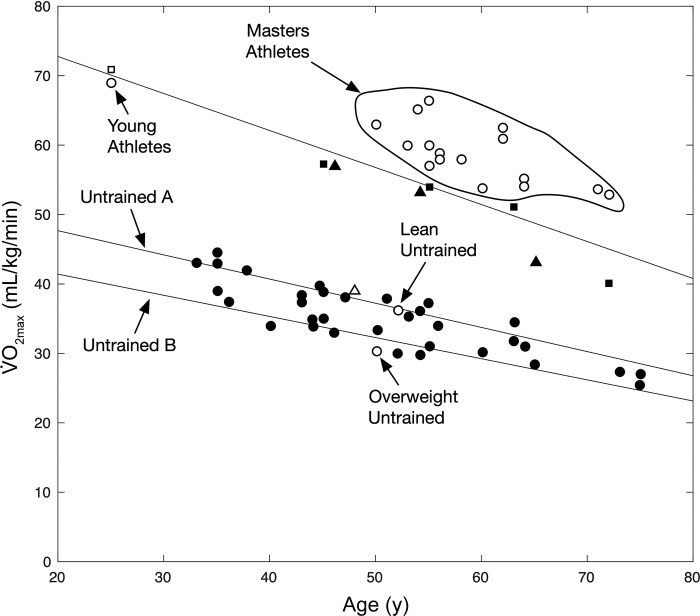
Decline in V̇o_2max_ with age in groups of sedentary and exercise-training men and the masters athletes. Individual data points are average V̇o_2max_ values for groups of men of different ages from 13 reports in the literature and for young athletes, masters athletes, lean untrained, and overweight untrained men in Heath et al. (70). [Reproduced from Heath et al. (70) with permission.]

The initial landmark study by the Holloszy laboratory on this topic was published in 1981 by Greg Heath (70). They used a clever version of the masters athlete model in which young and masters male endurance athletes were matched for training program and body weight, while age-matched older untrained subjects were matched for body composition. The masters athletes had an average V̇o_2max_ that was 15% lower than the young athletes (Fig. 14), but 100% higher than healthy untrained men, which was reduced to 60% after accounting for differences in lean body mass. The athlete groups also had a greater left ventricular (LV) volume and mass than the untrained controls. The masters athletes’ maximal heart rates were ~15% lower than the young athletes, but the maximal O_2_ pulse (V̇o_2max_/maximal heart rate) was similar for these two groups. This study demonstrated that V̇o_2max_ declines with aging in healthy adults, independent of physical activity and changes in body composition; however, at both young and older ages, endurance exercise-trained individuals maintain markedly higher levels of cardiorespiratory fitness compared with their sedentary peers. The findings also indicated that a decrease in maximal heart rate is a key mechanism in the decline in V̇o_2max_ with aging in healthy endurance-trained individuals.

To gain further insight into the mechanisms underlying decreases in V̇o_2max_ with aging, Hagberg and colleagues (60) assessed stroke volume during fixed relative (% maximal) intensities of submaximal exercise combined with measurements of heart rate during maximal exercise to estimate maximal exercise cardiac output and arteriovenous O_2_ difference (a-vO_2_diff). They found that estimated maximal stroke volume and a-vO_2_diff did not differ between training-matched groups of young athletes versus masters athletes, again pointing to a reduced maximal heart rate-driven reduction in maximal cardiac output as the primary mechanism for the decline in V̇o_2max_ with age in trained individuals. In contrast, the higher V̇o_2max_ of the masters athletes compared with age-matched sedentary adults was associated with greater estimated maximal stroke volume, cardiac output, and a-vO_2_diff.

The Holloszy laboratory extended this work with two papers published in the early 1990s. Using a more direct measurement of maximal cardiac output (acetylene rebreathing technique), Takeshi Ogawa and colleagues (136) reported that decreases in maximal stroke volume contribute along with reductions in maximal heart rate to the decrease in maximal cardiac output and V̇o_2max_ with aging in both sedentary and exercising men and women. Moreover, they found that decreases in maximal a-vO_2_diff contribute to the decline in V̇o_2max_ with aging in sedentary but not exercising individuals. The results also confirmed and extended the original findings of Heath et al. that age-related decreases in V̇o_2max_ in both men and women cannot be explained by changes in body composition (loss of lean mass and increases in body fatness). An analysis of skeletal muscle biopsies from these subjects performed by Andy Coggan and colleagues (21) showed that muscle oxidative capacity and capillary density decreased with aging in the sedentary individuals, but not in the endurance athletes. These latter observations provided evidence supporting the idea that, in contrast to sedentary adults, the maintenance of maximal a-vO_2_diff with aging in endurance athletes may be mediated, at least in part, by preservation of capillarity, mitochondria, and other adaptations in the trained muscles that support O_2_ conductance and utilization.

Finally, using a longitudinal study design, Marc Rogers and colleagues in the Holloszy laboratory (154) provided complementary insight to these cross-sectional investigations by reevaluating groups of masters endurance athletes and sedentary age-matched men after an average 8-yr follow-up period. They found that the masters athletes, all of whom had continued training during the follow-up period, demonstrated only ~50% of the decline in V̇o_2max_ as the sedentary men, which was associated with a maintenance of maximal heart rate in contrast to the decreases observed over time in the sedentary individuals. These findings support the concept that continued endurance training may, at least over a period up to a decade, mitigate a portion of the decrease in V̇o_2max_, in part by maintaining maximal heart rate.

### Exercise Training and Cardiorespiratory Fitness with Aging

Prior to the early 1980s, several laboratories had reported finding little or no change in V̇o_2max_ in response to aerobic exercise training in healthy older adults (e.g., >60 yr), in contrast to the clear improvements documented in younger groups (7, 31, 133, 134, 171). This led to the popular opinion that with aging the ability to adapt to exercise training with an increase in cardiorespiratory fitness was lost. Challenging conventional wisdom, as he often did, Holloszy hypothesized that previous findings in older adults were primarily the result of an inadequate exercise training stimulus (insufficient intensity, frequency, duration, and/or length of training).

In an initial test of this hypothesis published in 1984 (164), Doug Seals in the Holloszy laboratory conducted a two-phase aerobic exercise training study in a small group (*n* = 14) of healthy men and women aged 60–67 yr with a corresponding group of nonexercising controls. The exercising subjects performed lower-intensity training (brisk walking 4–5 days/wk) for 6 mo followed by an additional 6 mo of higher-intensity training (jogging or vigorous stationary cycling ≥3 days/wk). The lower-intensity training increased V̇o_2max_ by an average of 11%, which was equal to or greater than that reported previously in this age group. Importantly, the additional 6 mo of higher-intensity training produced a significant further increase in V̇o_2max_ such that the mean increase at 12 mo was 30%—a response 3- to 8-fold greater than previously reported. The exercising older adults also demonstrated CV adaptations to submaximal exercise that were similar to those reported previously in younger groups, including increases in stroke volume and reductions in heart rate, blood pressure, and peripheral vascular resistance at the same absolute workload versus before training. This was the first study to demonstrate that healthy older adults retain the ability to adapt to endurance exercise training with relative increases in V̇o_2max_ at least as great as those induced in younger adults.

The results of the original exercise intervention trial of Seals et al. (164) were later confirmed and extended in a major follow-up exercise intervention study performed by the Holloszy laboratory as part of an NIH Program Project. A paper published in 1991 by Wendy Kohrt and colleagues (112) showed a mean increase in V̇o_2max_ of 24% in response to 9–12 mo of vigorous aerobic exercise training in a much larger cohort of healthy older adults (*n* = 57F/53M; 60–71 yr). Importantly, they found no differences in the training-associated increases in V̇o_2max_ between men and women, nor were the increases in V̇o_2max_ related to subject age or baseline level of fitness.

In a second paper from this exercise trial, Bob Spina and colleagues (169) reported that the CV determinants of the increases in V̇o_2max_ in response to training differed between men and women. They found that the older men increased V̇o_2max_ via increases in maximal stroke volume and cardiac output (66% of the increase), with the remaining improvements associated with increases in maximal a-vO_2_diff. These adaptations were similar to those observed previously in younger men and women. In contrast, Spina et al. found that older women did not increase maximal stroke volume and cardiac output; thus their improvements in V̇o_2max_ were due solely to increases in maximal a-vO_2_diff.

In a third complementary paper from this study, Coggan et al. (19) determined if the increases in maximal a-vO_2_diff observed were associated with adaptations in the trained skeletal muscles. Analyzing muscle biopsy samples, in contrast to the findings of earlier studies, Coggan and colleagues found robust increases in trained skeletal muscle capillary density (~20%) and mitochondrial oxidative enzymes (25–55%). They concluded that skeletal muscles of older men and women retain the ability to undergo the same adaptations to training as young adults, provided the training stimulus is adequate, and that these adaptations may contribute to improvements in peripheral O_2_ utilization with training.

The Holloszy laboratory made many other important contributions to the field of exercise training and CV aging. In the late 1990s and early 2000s, Ellen Binder, Ali Ehsani, and Holloszy conducted the first studies to determine the effects of a multidimensional exercise program (sequential flexibility, stretching, light resistance exercise, and aerobic exercise activities) in community-dwelling adults in their 80s with mild to moderate frailty (9, 44). They found that this program increased V̇o_2peak_ by 13–14% in this group as a result of either increases in peak cardiac output alone (older men) or increases in both peak cardiac output and a-vO_2_ diff (older women). These studies demonstrated that mildly frail octogenarians could adapt to exercise training, albeit perhaps with a smaller capacity to improve maximal aerobic exercise capacity than younger groups.

### Exercise Training and CV Function in Patients with CAD

By 1980, the results of animal studies clearly demonstrated that endurance exercise training reduced myocardial ischemia, increased myocardial blood supply, and improved LV performance (71, 123, 193). However, although previous studies in patients with CAD had consistently shown improvements in aerobic exercise capacity with endurance training, the evidence suggested that these improvements were mediated by “peripheral adaptations” in the autonomic nervous system, peripheral vasculature, and skeletal muscle, and not to improved cardiac (LV pump) function per se (36, 37, 72, 135). As with his work in aging, Holloszy hypothesized that the failure of previous studies in patients to show direct adaptations to the heart was the result of an inadequate exercise training stimulus.

Accordingly, with the leadership of cardiologist Ali Ehsani, the Holloszy laboratory tested this hypothesis by conducting a major intervention study employing 10–12 mo of endurance exercise training of progressively increasing intensities not used previously in these patients. The results of these studies transformed thinking about the impact of exercise training on cardiac function in patients with CAD in a manner that still resonates today.

**Fig. 15.**
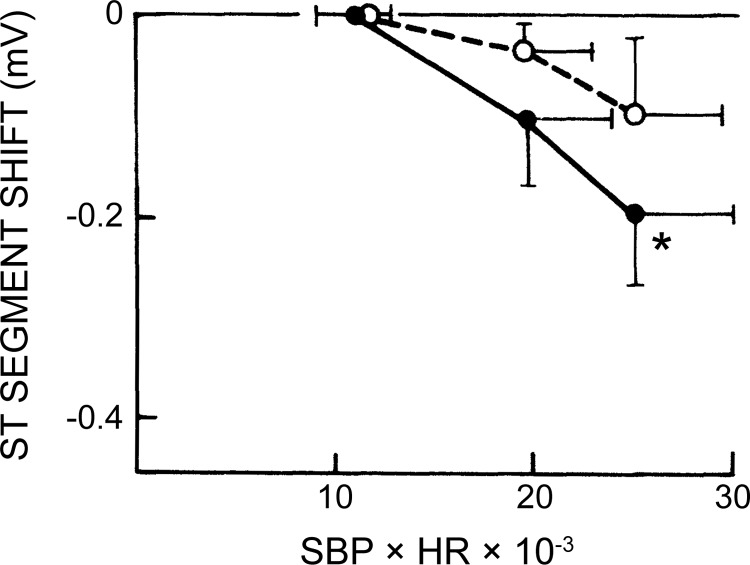
The relationship between the extent of ST-segment depression and the double product at different intensities of exercise before (solid circle and line) and after (open circle, dashed line) 12 mo of exercise training in coronary artery disease (CAD) patients. SBP, systolic blood pressure; HR, heart rate. Values are means ± SD. *Before training vs. after training, *P* < 0.02. [Reproduced from Ehsani et al. (42) with permission. Copyright American Heart Association.]

In two of the initial papers from this study, Ehsani et al. (42, 43) demonstrated that this prolonged and vigorous program of endurance exercise training improved true V̇o_2max_ (not V̇o_2peak_) by 40% in patients with CAD. Importantly, they also found that ECG-derived ST-segment depression, a measure of myocardial ischemia, during maximal exercise was reduced by 20% after training despite attaining a 20% greater maximal “double product,” an indicator of myocardial O_2_ demand, with associated higher maximal plasma norepinephrine levels, an indicator of sympathetic nervous system activity linked to greater myocardial O_2_ requirements (Fig. 15). These findings strongly suggested reduced myocardial ischemia at the same or even higher myocardial O_2_ demand after training.

These observations were supported and extended in a paper by Hagberg et al. (62) showing that this exercise training program improved stroke volume and “stroke work” (stroke volume at a particular blood pressure or “resistance” to LV contraction) in these patients during submaximal exercise, again suggesting cardiac rather than solely peripheral adaptations to training. Wade (Chip) Martin working in the Holloszy laboratory then used the assessment of systolic time intervals, another measure of myocardial function, to provide additional evidence for direct effects of training on the heart (122). This analysis was followed by a key paper in which Ehsani and colleagues (41) employed state-of-the-art imaging technology to demonstrate that the apparent improvements in LV function observed in their previous reports were, indeed, likely mediated by increases in LV contractility, as shown by the increase in ejection fraction from rest to maximal exercise after accounting for cardiac loading conditions. This finding provided more direct evidence that prolonged, vigorous endurance training reduces the severity of myocardial ischemia in these patients despite an increase in myocardial O_2_ requirement induced by acute exercise.

Finally, Marc Rogers and colleagues (156) showed that patients who completed the original 12-mo training program, but continued to exercise for an additional 6 yr, maintained their initial improvements in V̇o_2max_ and maximal ST-segment depression, and further improved their plasma HDL-C concentrations. This follow-up study demonstrated for the first time that the beneficial effects of intensive initial training can be preserved for long periods in motivated patients who continue to exercise.

### Conclusions

Holloszy and his laboratory have made physiologically and clinically important contributions to the field of CV exercise physiology, in particular by demonstrating the clear beneficial effects of exercise training for improving V̇o_2max_ and CV function in both older healthy adults and in patients with CAD.

## HUMAN GENERAL EXERCISE PHYSIOLOGY STUDIES

In addition to Holloszy’s pioneering studies in the exercise-related disciplines outlined above—skeletal muscle, diabetes, aging, caloric restriction, and the CV system—the continual flow of research exercise physiologists through his laboratory provided a fertile environment for a wide range of studies that also addressed numerous wide-ranging but critical aspects of basic human exercise physiology. A sampling of these contributions is summarized below.

### Carbohydrate Feedings During Exercise

In 1981, Fanie Terblanche et al. (173) in the “animal laboratory” reported that glycerol feedings in rats markedly increased their endurance capacities. Soon thereafter, independent studies in Holloszy’s “upstairs” human laboratory as well as in Dave Costill’s laboratory at Ball State found that glycerol feedings failed to increase endurance in humans (127). However, this raised the question as to whether glucose or maltodextrin feedings might improve endurance in humans, which had been addressed decades earlier. Indeed, Coyle et al. found that carbohydrate feeding significantly delayed the time of fatigue, defined as a 10% drop in initial power output, from 134 to 157 min (23). It was initially postulated that the mechanism for delayed fatigue might be muscle glycogen sparing (23), yet later studies found no glycogen sparing and concluded that the direct oxidation of the ingested glucose was the mechanism for delayed fatigue (22).

### Anaerobic Threshold and McArdle’s Disease Patients

A portion of the Holloszy “upstairs” human laboratory was supported by the Neuromuscular Disease Clinic at the Washington University School of Medicine, and exercise tests on a wide range of these patients were performed on a regular basis. This led to the realization that this clinic would have access to a specific population, McArdle’s disease patients, that could potentially be useful in addressing indirectly the mechanistic basis for the “anaerobic threshold,” which at the time was a very hot topic in exercise physiology research (182). Since these individuals lack muscle phosphorylase, they cannot process glucose molecules rapidly enough to raise blood lactate levels. In fact, the first diagnostic assessment for this disease was to have them perform muscle handgrip exercise with a cuff on their upper arm inflated to suprasystolic levels. Patients with this disease experienced no increase in blood lactate levels during such a challenge, while healthy individuals experienced substantial blood lactate increases. Clearly, according to current dogma at the time, if the increase in ventilation at ~60–75% V̇o_2max_ during an incremental exercise test was the result of increases in lactate production, these patients should not elicit such a threshold with progressive exercise. However, two studies in the Holloszy laboratory by Hagberg et al. and subsequently by others indicated that the patients did, in fact, have such a “breakpoint” in their ventilation and proposed it should be termed more descriptively as the “ventilatory threshold” (61, 63).

### Exercise Training Effects on V̇o_2max_ in Humans

**Fig. 16.**
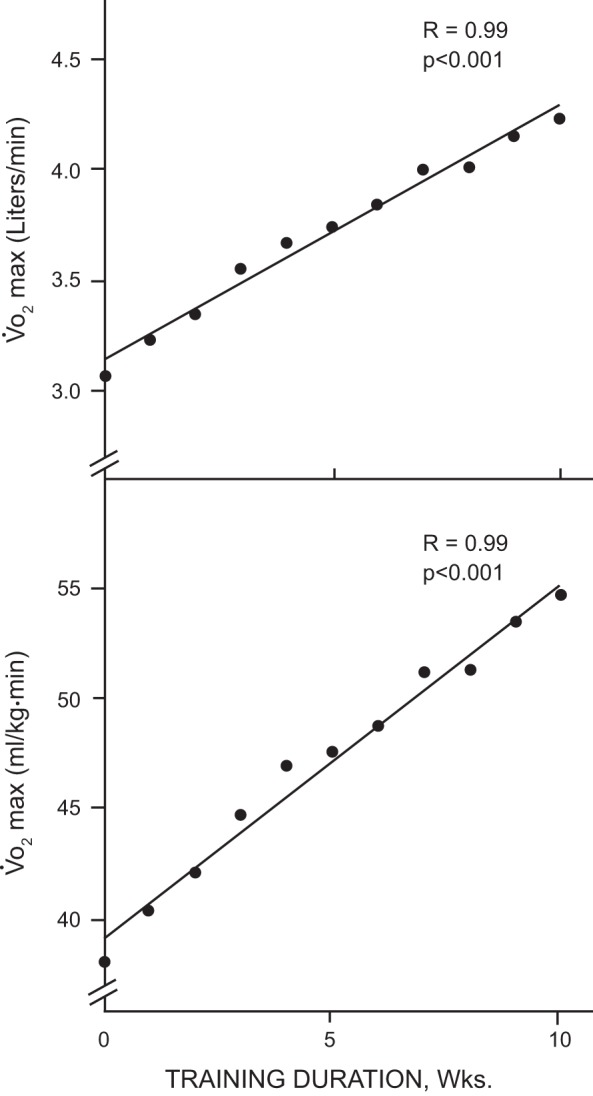
Increases in subjects’ average V̇o_2max_ during the 10-wk intensive Hickson training program. [Reproduced from Hickson et al. (75) with permission.]

**Fig. 17.**
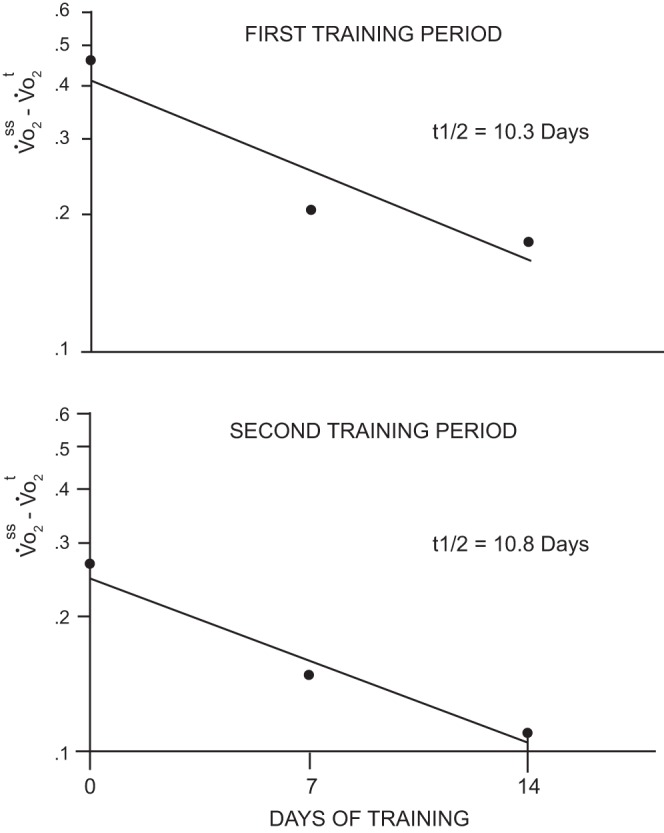
Semilogarithmic plots of the increase in V̇o_2max_ during two training periods utilizing the Hickson training protocol. Values plotted are the differences between the new, higher steady state (V̇o_2_^ss^) and the values at given times during training (V̇o_2_^t^). [Reproduced from Hickson et al. (76) with permission from Wolters Kluwer Health, Inc. Copyright American College of Sports Medicine.]

At the time in the mid 1970s it was generally believed that individuals could, at most, increase their V̇o_2max_ by up to ~25% with exercise training. Following the philosophy that more intense and prolonged exercise training may elicit greater improvements in V̇o_2max_ than those previously observed, Bob Hickson et al. (75) employed high-intensity interval training on the cycle ergometer (long before the current HIIT rage) along with prolonged intense running on alternate days, for a total of 6 days/wk of training over 10 wk. They found linear improvements in V̇o_2max_ continuing over the 10-wk training program amounting to individual increases of 17–58% with the average increase being 44% (Fig. 16). This was one of the first studies to employ interval training, common to athletes, to demonstrate that aerobic capacity in untrained people can increase quickly and to a much greater extent than previously thought possible. They also went on to show that the half-time of the V̇o_2max_ increase was ~10 days and that further adaptations require progressive increases in exercise intensity (76) (Fig. 17). These observations are a hallmark of the concept of “periodization of training.”

### Exercise Training-Induced Hormonal Changes

Given the strong interests of the Holloszy laboratory in hormonal responses to acute exercise and exercise training, it is hardly surprising that a number of important papers in this area were also published from the laboratory, generally under the direction of Will Winder. In one of the intense-training studies, participants also underwent two additional weekly exercise tests to assess the time course of the adaptations of hormonal responses to acute exercise with training. For the first study, participants performed a 5-min cycle ergometer bout at 95% V̇o_2max_ on a weekly basis which showed that roughly half of the reductions in plasma epi- and norepinephrine elicited over the entire 7-wk training program were evident after only the first week of training, whereas the heart rate and blood lactate reductions took place over a slightly longer time course (186). In a second study in the same individuals, plasma epi- and norepinephrine and glucagon responses to a 90-min exercise bout at ~60% V̇o_2max_ decreased substantially over the course of the 9-wk training program; however, again the overwhelming majority of the adaptation took place after only 3 wk of training, the first time point of measurement (188). Thus these data clearly show, again, that many of the adaptations to long-term training actually occur to a substantial extent in only the initial weeks of training.

### Endurance Training and Metabolism in Healthy Individuals

In the 1980s it was known that exercise training resulted in an increased reliance on fat as a substrate during exercise, with a concomitant sparing of muscle glycogen. However, the actual source of the increased fat oxidation was not clear: was it the result of increased plasma free fatty acid (FFA) or intramuscular triglyceride (TG) utilization? To further study these metabolic effects of endurance exercise training, Hurley et al. (94) with the help of Patti Nemeth assessed the decline in intramuscular triglyceride concentration during a 120-min bout of moderate-intensity exercise (64% of initial V̇o_2max_) performed at the same absolute power output before and after 12 wk of the Hickson high-intensity training program described above. The training program resulted in a 26% increase in V̇o_2max_ and, as expected, fat oxidation was higher in the trained state. However, the new finding was that intramuscular TG use roughly doubled with training. During this same exercise bout, Martin et al. also showed that plasma FFA turnover and oxidation were actually decreased after training (121). These observations made the important discovery in humans that the source of the well-known endurance training-induced increase in fat oxidation is intramuscular (intermyocellular) TG and not increased plasma FFA oxidation.

In another study from the Holloszy laboratory Andy Coggan et al. (17) studied plasma glucose turnover and oxidation also before and after training with measurements again made at a constant power of 60% pretraining V̇o_2peak_. They observed reduced rates of plasma glucose turnover and oxidation when trained, agreeing with a general sparing of carbohydrate. In a study of endurance-trained individuals matched for V̇o_2max_, glucose turnover and oxidation during exercise was 17–25% lower in subjects with a high lactate threshold (18). Interestingly, the percentage of total energy derived from glucose oxidation was inversely related to muscle citrate synthase activity (*r* = −0.85) supporting the concept developed by Holloszy that skeletal muscle oxidative capacity plays a major role in determining the metabolic response to submaximal exercise. The presence of more mitochondria after training reduces metabolic stress, thus reducing even glucose uptake by the active muscle.

With 12 wk of Hickson-type intense training Coggan et al. (20) observed increased mitochondrial activity and a lower glucose-6-phosphate concentration in muscle during exercise, suggesting that the classic reduction in carbohydrate utilization when trained results from attenuation of flux before the phosphofructokinase (PFK) step in glycolysis and is not due to citrate-mediated inhibition of PFK as proposed by Randle et al. (145).

### Effect of Exercise Training on Bone Mineral Density

Gail Dalsky et al. from the Holloszy laboratory published one of the early studies on the effects of exercise training on bone mineral density (BMD) in older otherwise healthy women (30). They posited that these women might not be taking in enough Ca^2+^ to improve their BMD, and thus all participants took Ca^2+^ supplements across the trial. The exercise training intervention was developed to impart significant skeletal loading and primarily consisted of treadmill exercise and loaded stair-walking. Following 9 mo of this intervention BMD increased by an average of 5.2% in the intervention group, which was significantly greater than the slight reduction evident in the control group over that time. Furthermore, with another 13 mo of training, thus 22 mo in total, BMD in these women had increased by 6.1%. As another clear demonstration of the direct effect of exercise training on BMD, these women then lost nearly all of their training-induced increase in BMD following a 13-mo period when they stopped the training. These were some of the first BMD measurements from a prolonged targeted exercise training intervention that also incorporated a control group and then also a period of detraining following training.

### Resistance Exercise Training

Hurley et al. (95) in the Holloszy laboratory had middle-aged men perform heavy resistance training for 16 wk and observed a 44% increase in strength with no improvement in V̇o_2max_. In another study, it was reported that the training regimen of bodybuilders is associated with a healthier blood lipid profile than seen in powerlifters (93). Furthermore, androgen use by strength-trained athletes appeared to worsen the blood lipid profile and increase the risk for coronary heart disease (96). The work by Ben Hurley in the Holloszy laboratory and subsequently at the University of Maryland made it very clear that resistive exercise training had the potential to lower the risk of coronary disease without altering V̇o_2max_ (92).

### Detraining

**Fig. 18.**
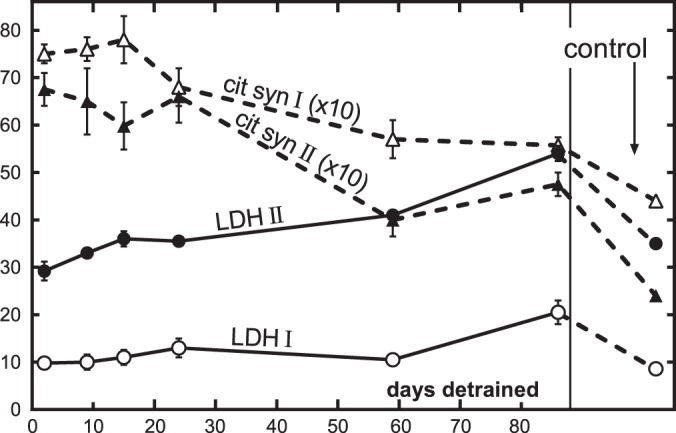
Sequences of detraining changes in citrate synthase (cit syn) and lactate dehydrogenase (LDH) in type I and type II vastus lateralis fibers from cyclist A (Holloszy). Fiber types are indicated by I and II. Each point is average ± SE for 6–23 fibers in the case of LDH and 6–16 fibers (chosen from the same groups) in the case of citrate synthase. Control indicates average for vastus lateralis fibers from both controls. Activities are in mol·kg dry wt^−1^·h^−1^ at 20°C. Note scale for citrate synthase is 0–8 instead of 0–80. [Reproduced from Chi et al. (16) with permission.]

In the early 1980s, Holloszy and his group were surprised by reports that V̇o_2max_ and other adaptations decline rapidly when endurance athletes stop training, and they sought to examine this further. Although it was difficult to find endurance athletes willing to stop training, a number of the laboratory’s scientists (many of whom were coauthors on those and this paper), including Holloszy (16) and four competitive runners and two cyclists, who had trained intensely for years, then stopped training for 3 mo (26). Indeed, V̇o_2max_, maximal cardiac output, and oxidative enzyme activity all declined with a half-life of 10–14 days, whereas SV declined even more rapidly, which was later found to be due to a decrease in blood volume (24). Oliver Lowry collaborated on these studies and applied his remarkable single muscle fiber enzyme analysis techniques to differentiate the enzymatic changes in Type I and Type II muscle fibers (16). Oxidative enzyme activity when trained was approximately equal in Type I and Type II muscle fibers due to the high-intensity interval training before detraining. Furthermore, oxidative enzyme activity declined in both fiber types with a half-life of ~12 days in the group, while Holloszy showed a delayed drop in citrate synthase in Type I fibers (Fig. 18). Although the level in the Type I fibers approached that of untrained control subjects, the oxidative activity of the Type II fibers remained ~80% above untrained control levels and only ~10–20% lower than the level in detrained Type I fibers (16, 25, 26, 77). Thus oxidative capacity in Type II muscle fibers was retained above untrained levels (16, 25, 26, 77, 165). Additionally, the three CV factors that were fully retained at trained levels after ~2–3 mo of detraining were muscle capillary density (25, 26), and end-diastolic volume and stroke volume while exercising in the supine position, which augments ventricular filling (120, 122). Therefore, there was little evidence that intrinsic heart size or function is reduced with 2–3 mo of detraining.

Thus, although the studies summarized in this section may not necessarily have addressed the primary foci of the career of John Holloszy—skeletal muscle, diabetes, aging, caloric restriction and the CV system—they also made substantial advances in our knowledge of basic human exercise physiology and many are considered classics in their areas.

## QUANTITATIVE EVIDENCE OF HOLLOSZY’S HISTORICAL IMPACT

Clearly, John Holloszy has had a substantial impact on the thinking and the scientific progress of a number of disparate research foci within the areas of human and animal exercise biochemistry and physiology. One major factor underlying this impact is the 410 manuscripts that he has published over the course of his career, manuscripts that have been published in a wide range of general and specialty journals, including the *Journal of the American Medical Association;* *Proceedings of the National Academy of Sciences USA;* *Science;* *Nature Medicine;* *Journal of Clinical Investigation;* *Journal of Biological Chemistry;* *Annals of the New York Academy of Science;* *Circulation;* *Circulation Research;* *Acta Medica Scandinavica;* *Diabetes;* *Diabetes Care;* *Acta Physiologica Scandinavica;* *Cell Metabolism;* *Arteriosclerosis*, *Thrombosis* *and Vascular Biology;* *American Journal of Physiology;* *Journal of Applied Physiology;* and *Medicine and Science in Sports and Exercise*, among others. And, as the coauthors of the present paper can attest, his name was not just “added” to the author list. In fact, often he took his name off of manuscripts that he felt he had not contributed to substantially.

Further evidence of the quality of these publications is that they have been cited ~41,000 times as of April 1, 2018. To put this into perhaps a somewhat more comprehensible context, this translates to two scientists every day citing Holloszy’s publications since the day he published his first manuscript in 1961 until the present. In comparison, it appears that only three other exercise scientists have achieved these levels of citations over the course of their careers for original data and review papers. Furthermore, 10 of Holloszy’s postdoctoral trainees own papers that have been cited >10,000 times.

In terms of individual papers, Holloszy’s 1967 paper in the *Journal of Biological Chemistry* titled “Biochemical adaptations in muscle: Effects of exercise on mitochondrial oxygen uptake and respiratory enzyme activity in skeletal muscle” has been cited ~1,050 times (78) (Table 3). His review paper with Ed Coyle in the *Journal of Applied Physiology* (82) in 1984 titled “Adaptations of skeletal muscle to endurance exercise and their metabolic consequences” has been cited ~1,250 times. And his review article with Frank Booth in 1976 titled “Biochemical adaptations to endurance exercise in muscle” in the *Annual Review of Physiology* (81) has been cited ~1,000 times. In addition, Holloszy has a total of 48 publications that have been cited >200 times and 138 publications that have been cited >100 times to date. Overall, John Holloszy’s ISI h-index is 118, indicating that he has 118 papers which each have been cited more than 118 times. While perhaps not stacking up that well with Sigmund Freud, the individual with supposedly the highest h-index (272 h-index), Holloszy’s h-index is among the highest in the general discipline of exercise sciences.

**Table 3.** Holloszy most-cited articles from online Web of Science

Total Citations*	Authors	Title	Reference
1,259	Holloszy JO, Coyle EF	Adaptations of skeletal muscle to endurance exercise and their metabolic consequences	*J Appl Physiol* 56: 831–838, 1984 (82)
1,068	Holloszy, JO	Biochemical adaptations in muscle – effects of exercise on mitochondrial oxygen uptake and respiratory enzyme activity in skeletal muscle	*J Biol Chem* 242: 2278–2282, 1967 (78)
982	Holloszy JO, Booth FW	Biochemical adaptations to endurance exercise in muscle	*Annu Rev Physiol* 38: 273–291, 1976 (81)
613	Baar K, Wende AR, Jones TE, Marison M, Nolte LA, Chen M, Kelly DP, Holloszy JO	Adaptations of skeletal muscle to exercise: rapid increase in the transcriptional coactivator PGC-1	*FASEB J* 16: 1879–1886, 2002 (4)
598	Leone TC, Lehman JJ, Finck BN, Schaeffer PJ, Wende AR, Boudina S, Courtois M, Wozniak DF, Sambandam N, Bernal-Mizrachi C, Chen Z, Holloszy JO, Medeiros DM, Schmidt RE, Saffitz JE, Abel ED, Semenkovich CF, Kelly DP	PGC-1 alpha deficiency causes multi-system energy metabolic derangements: Muscle dysfunction, abnormal weight control and hepatic steatosis	*PLoS Biol* 3: 672–687, 2005 (117a)
498	Winder WW, Holmes BF, Rubink DS, Jensen EB, Chen, M, Holloszy JO	Activation of AMP-activated protein kinase increases mitochondrial enzymes in skeletal muscle	*J Appl Physiol* 88: 2219–2226, 2000 (188a)
475	Baldwin KM, Klinkerfuss GH, Terjung RL, Mole PA, Holloszy JO	Respiratory capacity of white, red, and intermediate muscle: adaptive response to exercise	*Am J Physiol* 222: 373–378, 1972 (5)
473	Fontana L, Meyer TE, Klein S, Holloszy JO	Long-term calorie restriction is highly effective in reducing the risk for atherosclerosis in humans	*Proc Natl Acad Sci USA* 101: 6659–6663, 2004 (49)
442	Douen AG, Ramlal T, Rastogi S, Bilan PJ, Cartee GD, Vranic M, Holloszy JO, Klip A	Exercise induces recruitment of the “insulin-responsive glucose transporter”. Evidence for distinct intracellular insulin- and exercise-recruitable transporter pools in muscle	*J Biol Chem* 265: 13427–13430, 1990 (38)
414	Hurley BF, Nemeth PM, Martin WH, Hagberg JM, Dalsky GP, Holloszy JO	Muscle triglyceride utilization during exercise: effect of training	*J Appl Physiol (85)* 60: 562–567, 1986 (94)

* Total citations as of May 5, 2018.

In terms of publications in American Physiological Society journals, Holloszy has 86 publications in the *Journal of Applied Physiology,* and these publications have been cited ~11,000 times with 38 (nearly half of his *Journal of Applied Physiology* articles) being cited >100 times. In fact, Holloszy has an h-index of 56 based only on his *Journal of Applied Physiology* publications—an h-index most investigators would love to have for their entire career, much less based only on publications from a single journal! Holloszy also has another 99 publications in the different components of the *American Journal of Physiology*, and these articles have been cited a total of ~8,700 times resulting in an h-index of 56 with 26 *American Journal of Physiology* articles cited >100 times. Between the *Journal of Applied Physiology* and the *American Journal of Physiology*, Holloszy has a total of 185 publications, a total of ~20,000 citations, an h-index of 79, and 64 papers that have been cited >100 times each.

This was all accomplished as a result of numerous grants to support Holloszy’s research and that of his postdoctoral fellows over the 50+ years of his career. In fact, in terms of only NIH, Holloszy had a total of ~150 yr of funding, which averages to his virtually having three large NIH grants funded concurrently across his entire career (Table 4). The three standouts on this NIH grants list are *1*) R01 funding by National Institute on Aging (NIA) for 48 consecutive years titled “Exercise-Induced Biochemical and Anatomical Adaptations”; *2*) a second R01 funded by National Institute of Diabetes and Digestive and Kidney Diseases for 32 years titled “Carbohydrate and Fat Metabolism During Exercise”; and *3*) an Institutional Postdoctoral Training Fellowship Grant (T32) funded for 24 years by NIA titled “Exercise as Preventive Medicine in the Aging Process.” In addition, from NIH Holloszy has also been funded by two other R01 grants, two Program Projects (P01), a P60 Comprehensive Center of Excellence, and a U01 Research Project Cooperative Agreement (Table 4). And this list does not include NIH grants obtained by others working in his laboratory or by his collaborators, nor any grants from other funding organizations!

**Table 4.** Holloszy NIH grant support

Grant Title	Grant Type	NIH Institute	Grant Length, yr
Exercise-Induced Biochemical and Anatomical Adaptations	R01	NIA	48
Carbohydrate and Fat Metabolism During Exercise	R01	NIDDK	32
Exercise as Preventive Medicine in the Aging Process	T32	NIA	24
Effect of Exercise Training in Ischemic Heart Disease	R01	NHLBI	8
Caloric Restriction and Aging in Humans	U01	NIA	7
Physiological Adaptations to Exercise in the Elderly	P01	NIA	10
Can Exercise Training Reverse Physical Frailty?	P60	NIA	6
Claude D. Pepper Older Americans Intervention Center	P60	NIA	6
Is DHEA Supplementation Beneficial?	R01	NIA	5

NIH, National Institutes of Health; NIA, National Institute on Aging; NIDDK, National Institute of Diabetes and Digestive and Kidney Diseases; NHLBI, National Heart, Lung, and Blood Institute.

## HOLLOSZY SCIENTIFIC FAMILY SECOND GENERATION

**Fig. 19.**
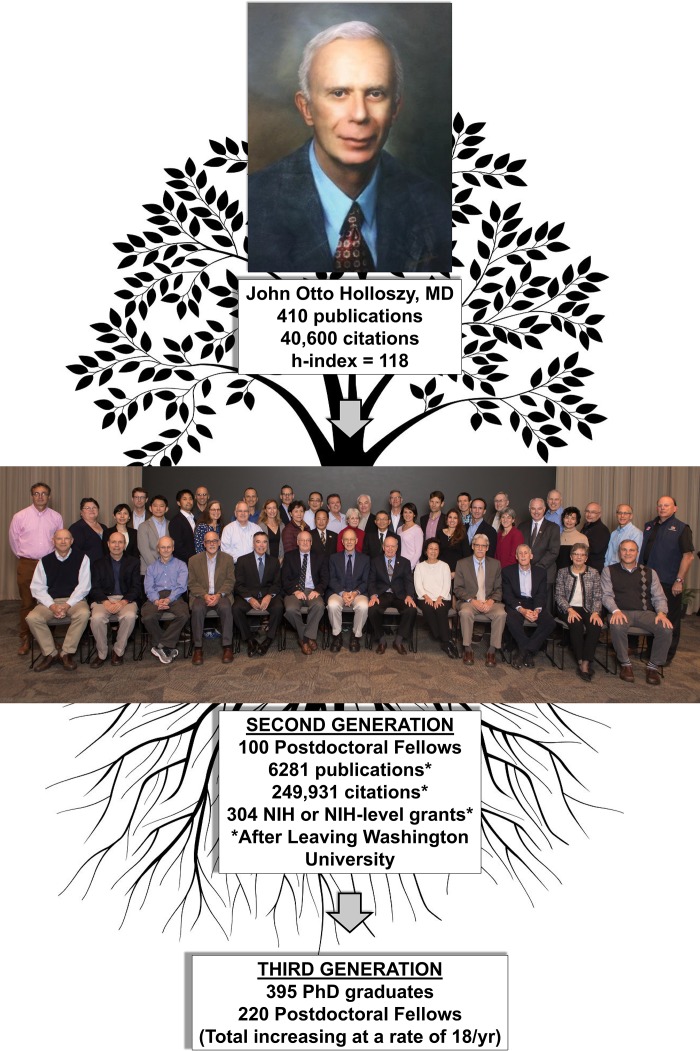
The three generations of the John O. Holloszy scientific family tree. Permission to use the picture of Dr. John Holloszy is provided from the Washington University School of Medicine Division of Geriatrics and Nutritional Science. [Photo in middle used with permission. Copyright Whitehall Photography (www.whitehallphotography.com).]

Virtually at the start of his career, Holloszy integrated postdoctoral research fellows into his research, with the first being Larry Oscai in 1967 followed by Paul Mole in 1969. This was not a model that was used previously in the discipline of exercise biochemistry/physiology. In fact, even during the 1970 and 1980s, rarely did an exercise biochemistry/physiology faculty position announcement mention the need or even desire for postdoctoral training for the applicant. Quite the opposite is the case now with almost all such faculty position announcements at major research universities requiring postdoctoral training for a viable candidate. As a result of Holloszy starting with postdoctoral fellows so early in his career and having such a long career, he directly supervised ~100 postdoctoral research fellows in his laboratories over the course of his career. Virtually all of them have gone on to make their own quite substantial marks on the field of exercise physiology/biochemistry with many of them mentioned above relative to their seminal publications (Fig. 19). The excellence of the training provided by John Holloszy is clearly demonstrated by the institutions where these individuals have been on faculty, which include Texas, Missouri, Colorado, Maryland, Brigham Young, Michigan, Duke, California-Irvine, Ball State, Michigan, Connecticut, Arizona, Southern California, Georgia, Mayo Clinic, Case Western, Georgia, Texas A & M, Karolinska Institute, Pennington Biomedical Research Center, and Washington University itself. The positions these individuals have held is also a testament to the training and preparation provided by John Holloszy, as they include professors, department chairs, deans, associate deans, chancellors, and presidents.

This second generation has also been massively productive in the field of exercise physiology/biochemistry as they have published ~7,500 research manuscripts in total, an average of ~6 publications/fellow in collaboration with Holloszy and an average of another 72 (~90% of their total publications) after they completed their training at Washington University. The quality of these publications is evidenced by the fact that in total they have been cited ~350,000 times, amounting to 17 citations every day from Holloszy or one of his direct trainees since the year of Holloszy’s first publication in 1961 until the present. Moreover, this Holloszy second generation has ~300 publications with Holloszy as a coauthor that have been each cited >100 times and another ~600 publications cited >100 times that were published from their own independent laboratories. Further evidence of the quality of the trainees supervised by Holloszy is the fact that this group has had >300 of their own NIH or NIH-level research grants over the courses of their careers, amounting to a total of ~1,300 individual years of grant funding!

## HOLLOSZY SCIENTIFIC FAMILY THIRD GENERATION

That the Holloszy scientific and historical legacy will continue far into the future is evidenced by the fact that his fellows themselves have to this point graduated ~430 PhD students and have supervised ~250 postdoctoral research fellows, numbers that both keep increasing by 5–10 new postdocs and PhD graduates every year. So, from the start with John Holloszy, the second direct scientific generation consisted of ~100 postdoctoral fellows, and the third generation added another nearly 700 scientists, and the number keeps increasing. Thus, in total across these three generations, ~800 high-level scientists have already been involved, keeping in mind, again, that this third generation continues to increase on a yearly basis.

## CONCLUSION

For almost 60 years John Holloszy maintained an intense and rigorous career where he searched for and found many answers to complex scientific questions in the area of exercise biochemistry and physiology. Very few, if any, prior scientists have made such substantive and critical contributions across such a wide continuum of scientific disciplines. How many scientists have ever attended such disparate national and international meetings as the American Diabetes Association, the Gerontology Society of America, Experimental Biology, and the American College of Sports Medicine and been recognized as a long-standing and critical research contributor to each and every one of these fields? The quantity and quality of his scientific contributions are clear—from the 400+ publications, the 41,000+ citations of these publications, and the numerous highly competitive grants he garnered to support his research over the course of his career. The quality of his research is most clearly documented by the seminal results he and his laboratory have produced over those 5+ decades in the areas of skeletal muscle biochemistry, skeletal muscle glucose uptake, mitochondrial biogenesis, caloric restriction, aging, CV disease, frailty, and general human exercise biochemistry and physiology.

Although it might be hard to imagine, even more important than all these seminal discoveries is Dr. Holloszy’s longstanding and massively productive mentorship of especially postdoctoral research fellows. His training of ~100 such fellows over the course of his career leaves another indelible mark on the field as they have virtually all gone on to make their own advances in the fields of exercise biochemistry and exercise physiology in both animal and human models. These fellows have trained the third generation of “Holloszy-bred” scientists, actually nearly another 700 of them, and growing each and every year. Thus it is clear that the disciplines of exercise biochemistry and exercise physiology, whether related to aging, frailty, diabetes, or disease prevention, will continue to be significantly impacted by the mind and science of John Holloszy for a long time into the future.

## GRANTS

G. D. Cartee was supported by National Institute of Health (NIH) Grants R01-DK-71771 and R01-AG-010026.

## DISCLOSURES

All authors acknowledge that they underwent previous research training under the direction of Dr. John O. Holloszy.

## AUTHOR CONTRIBUTIONS

J.M.H. conceived and designed research; J.M.H., E.F.C., K.M.B., G.D.C., L.F., J.P.K., D.R.S., and E.P.W. prepared figures; J.M.H., E.F.C., K.M.B., G.D.C., L.F., M.J.J., J.P.K., D.R.S., and E.P.W. drafted manuscript; J.M.H., E.F.C., K.M.B., G.D.C., L.F., M.J.J., J.P.K., D.R.S., and E.P.W. edited and revised manuscript; J.M.H., E.F.C., K.M.B., G.D.C., L.F., M.J.J., J.P.K., D.R.S., and E.P.W. approved final version of manuscript.
